# Traversability Assessment and Trajectory Planning of Unmanned Ground Vehicles with Suspension Systems on Rough Terrain

**DOI:** 10.3390/s19204372

**Published:** 2019-10-10

**Authors:** Kai Zhang, Yi Yang, Mengyin Fu, Meiling Wang

**Affiliations:** 1 School of Automation, Beijing Institute of Technology, Beijing 100081, China; kaizhangbit@gmail.com (K.Z.); fumy@bit.edu.cn (M.F.); wangml@bit.edu.cn (M.W.); 2 School of Automation, Nanjing University of Science and Technology, Nanjing 210094, China

**Keywords:** autonomous navigation, mobile robot, unmanned ground vehicle, light detection and ranging sensor, rough terrain

## Abstract

This paper presents a traversability assessment method and a trajectory planning method. They are key features for the navigation of an unmanned ground vehicle (UGV) in a non-planar environment. In this work, a 3D light detection and ranging (LiDAR) sensor is used to obtain the geometric information about a rough terrain surface. For a given SE(2) pose of the vehicle and a specific vehicle model, the SE(3) pose of the vehicle is estimated based on LiDAR points, and then a traversability is computed. The traversability tells the vehicle the effects of its interaction with the rough terrain. Note that the traversability is computed on demand during trajectory planning, so there is not any explicit terrain discretization. The proposed trajectory planner finds an initial path through the non-holonomic A*, which is a modified form of the conventional A* planner. A path is a sequence of poses without timestamps. Then, the initial path is optimized in terms of the traversability, using the method of Lagrange multipliers. The optimization accounts for the model of the vehicle’s suspension system. Therefore, the optimized trajectory is dynamically feasible, and the trajectory tracking error is small. The proposed methods were tested in both the simulation and the real-world experiments. The simulation experiments were conducted in a simulator called Gazebo, which uses a physics engine to compute the vehicle motion. The experiments were conducted in various non-planar experiments. The results indicate that the proposed methods could accurately estimate the SE(3) pose of the vehicle. Besides, the trajectory cost of the proposed planner was lower than the trajectory costs of other state-of-the-art trajectory planners.

## 1. Introduction

Recently, ground mobile robots with different functions start to play essential roles in people’s daily life. In particular, autonomous navigation in a non-planar environment is an important function. It enables competent operations of a ground mobile robot in many challenging applications, such as surveillance, rescue, and planet exploration. In this search field, two basic and critical problems need to be addressed: traversability assessment and trajectory planning. On the one hand, the traversability tells a robot the effects of its interaction with a rough terrain surface. The terrain information is obtained using a 3D light detection and ranging (LiDAR) sensor and/or a visual sensor. On the other hand, a trajectory planner utilizes a cost function considering the traversability to determine a dynamically feasible motion trajectory. This trajectory permits a robot to move from an initial pose to a goal pose. A pose is made up of the position (*x*, *y*, *z*) and the orientation (roll, pitch, yaw) of the robot.

On an uneven terrain surface, the motion of a ground robot is constrained by the terrain shape. Conventional trajectory planners that assume the terrain to be flat are not applicable, because the trajectories generated by them are hard to be tracked or even non-traversable. These trajectories can cause wheel slip, high roll/pitch angle, and so on. Therefore, it is necessary to quantitatively assess the utility of passing through an uneven terrain surface, and then generate a trajectory based on the utility. This utility is called traversability. Planning based on traversability can reduce the trajectory tracking error in a predictive way at the planning time, which otherwise is reduced using a feedback-based trajectory tracker at the control time.

Among different types of mobile robots, car-like unmanned ground vehicles (UGVs) are rapidly gaining popularity from researchers. A car-like vehicle is often equipped with a suspension system, which is the system of tires, tire air, springs, shock absorbers, and linkages that connects the vehicle to its wheels and allows relative motion between the two. The suspension system has a significant effect on the traversability of the vehicle. However, the suspension system was often neglected in previous work. Therefore, to generate a dynamically feasible trajectory, the proposed approach incorporates the model of the vehicle’s suspension system.

The purpose of this work is to navigate a car-like UGV safely and efficiently, with a focus on rough and unstructured terrain. The proposed methods can help an autonomous vehicle to be applied in many challenge applications. For example, the car-like vehicle may be required to dig a hole or unload something on a complex terrain surface. Besides, in the application of teleoperations, the proposed planner can be used to autonomously drive the vehicle to an operator-designated pose. This can reduce the workload of the operator and the telemetry bandwidth.

In summary, the contributions of this paper and the characteristics of the proposed methods are shown as follows:

1. The suspension system of the vehicle is used to reduce the pose estimation error and optimize the trajectory. The optimized trajectory is easy to be tracked by the vehicle in non-planar environments.
2. The traversability is assessed on demand based on original LiDAR points during trajectory planning, without any kind of explicit terrain surface reconstruction or discretization. This feature makes the proposed method efficient in terms of computation and storage.
3. The cost function and the node-expansion rule of the conventional A* are modified to obtain a path satisfying non-holonomic constraints. This path is then optimized by a constraint-aware optimizer based on a custom cost function. The final trajectory is smoother and more traversable than those generated by other state-of-the-art methods.
4. The proposed traversability assessor (or trajectory planner) is general and can be used with any other motion planning method (or traversability assessment method).

The rest of this paper is organized as follows. First, some previous researches about the traversability assessment and trajectory planning in a non-planar environment are reviewed. Then, Section 2 introduces the architecture of the proposed methods briefly. Section 3 describes how to assess the traversability of a ground vehicle based on its suspension system using a 3D LiDAR. Section 4 introduces how to generate and optimize a vehicle trajectory on rough terrain using the assessed traversabilities. Section 5 shows and analyzes the results of some simulation and real-world experiments. Finally, the paper is concluded and a direction for future work is suggested.

### 1.1. Related Work

The safe and efficient navigation of an unmanned ground vehicle requires the terrain information, which can be exploited to predict the future pose of the vehicle and assess the traversability. The sensors used to obtain the terrain information include visual sensors [1,2,3,4,5], LiDARs [6,7,8,9,10,11,12,13], and so on. Visual sensors can provide various kinds of terrain information, but processing visual data often requires a long computation time. What is more, the performance of visual sensors may vary with the intensity of sunlight [14]. The point clouds from LiDARs usually occupy large storage space, but the performance of a LiDAR-based terrain mapping approach is often relatively stable [15]. The point clouds or the visual data can be converted into traversabilities directly, or be converted into a digital elevation map (DEM) [16] or a 3D grid map [17,18]. A DEM is a 2.5D grid map with each grid value representing the height information about the terrain surface. It is easy to be implemented but cannot model overhanging obstacles, such as bridges. Besides, accurately computing the traversability needs a high resolution DEM, but building and maintaining a high resolution DEM is time-consuming. A 3D grid map is able to reconstruct an environment and suitable for the navigation in a multi-level building [19], but it is inefficient in terms of computation and storage. Compared with the existing methods, the proposed method assesses the vehicle traversability on demand using the original point cloud from a 3D LiDAR. It does not include complex terrain mapping process, so it is more efficient in terms of computation and storage.

A traversability assessor provides a mapping from the terrain maps or the sensor data to the traversabilities. It evaluates the mobility of a ground vehicle. The traversability depends on the pose of the vehicle and the terrain shape. The approaches that assess the traversability based on the terrain maps can be classified into appearance-based methods [20], geometry-based methods [21,22], and learning-based methods [23,24,25]. The approaches that assess the traversability based on original sensor data are often free of artificial discretization, which is inherent to DEMs, 3D grid maps, or other classical terrain models. For instance, Krüsi et al. propose an approach to determine the traversability of a specific pose without any discretization [26]. This approach can plan a trajectory directly on LiDAR points. Santamaria-Navarro et al. present a method for the large-scale traversability classification of point clouds [27]. In this method, the model for the classification of points is learned from training data using Gaussian processes. However, the existing methods usually ignore the vehicle’s suspension system.

Once the traversability is known, a trajectory should be generated. In the context of trajectory planning, researchers have paid much attention to the generation of a collision-free trajectory assuming flat terrain. Common trajectory planning techniques include artificial potential field (APF) methods [28,29], optimization-based methods [30,31], search-based methods [32,33], sampling-based methods [34,35], and so on. However, in a non-planar environment, more complicated cost functions and vehicle models must be used. For example, Amar et al. adapt the trajectory to the rough terrain using the kinematic model of the vehicle [36], and Howard et al. propose to optimize a trajectory by the model-based simulation of a vehicle on the rough terrain [37]. Recently, the trajectory planning methods based on machine learning are rapidly gaining popularity, especially the end-to-end learning methods. The main idea of these methods is to directly learn a mapping from the original sensor data to a trajectory. Learning-based planners have enabled a long-range autonomous navigation using a single stereo camera [38] or a LiDAR [39]. In terms of application, much of the work on trajectory planning in a non-planar environment focused on rovers for planetary exploration [40,41] or military vehicles [42].

The existing traversability assessors and trajectory planners seldom consider the suspension model of the vehicle. They usually take the whole vehicle as a rigid body. However, in a non-planar environment, the traversability of the vehicle largely depends on its suspension system. Therefore, the proposed methods incorporate the vehicle’s suspension model in traversability assessment and trajectory planning. This is essential for safe and efficient navigation in a non-planar environment.

## 2. System Architecture

**Figure 1:**
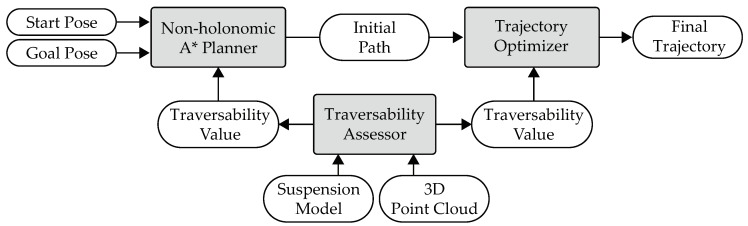
The architecture of the proposed traversability assessor and trajectory planner.

Figure 1 shows the architecture of the proposed traversability assessor and trajectory planner. The inputs of the system are a start pose and a goal pose. A non-holonomic A* planner generates an initial path, which is then optimized by a trajectory optimizer. Formally, a path is defined as the following mapping: [0,1]→X, and a trajectory is defined as the following mapping: [0,T]→X prescribing the evolution of the state of the vehicle in time, where *T* is the time instant at which the vehicle reaches the end of the trajectory, and X is the state space of the vehicle. The state of the vehicle can be defined as the pose (position and orientation) of the vehicle. The final output of the system is a solution trajectory. During the generation and the optimization of the trajectory, the traversability assessor is invoked by both the planner and the optimizer on demand to calculate the traversabilities at required poses. This calculation is based on a suspension model and the point clouds from a 3D LiDAR. Note that the traversability assessment is not treated as a separate and upstream process. It is regarded as an integral part of trajectory planning.

## 3. Traversability Assessment Using LiDAR

**Figure 2:**
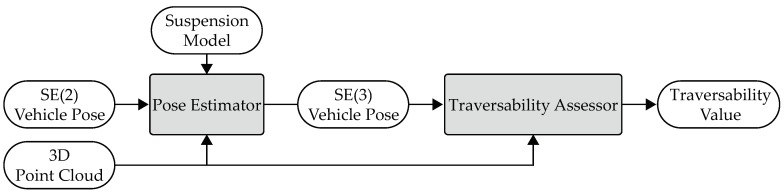
The overview of the traversability assessment approach.

In this section, a method will be introduced to assess the traversability of a vehicle using a 3D LiDAR. The traversability depends on the pose of the vehicle, the terrain roughness, and the height difference. The vehicle pose is estimated based on the point cloud and the suspension system. The overview of this section is shown in Figure 2.

### 3.1. Light Detection and Ranging

**Figure 3:**
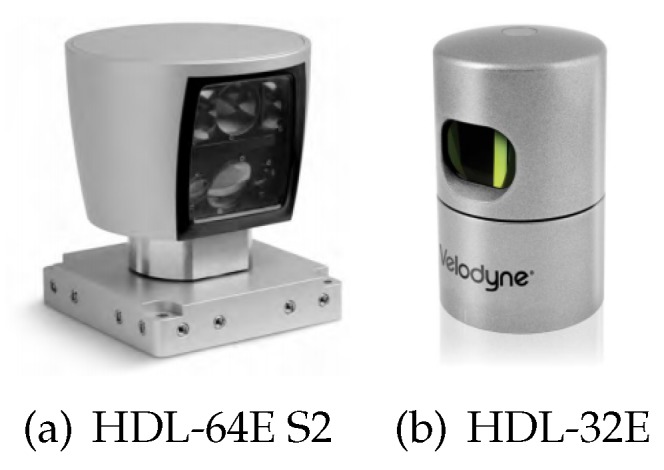
The 3D light detection and ranging (LiDAR) used in this work.

In this work, the 3D LiDAR used to acquire the geometric information of rough terrain is HDL-64E S2 or HDL-32E developed by Velodyne LiDAR, as shown in Figure 3. The manufactory is located at Silicon Valley. The specifications of the LiDAR are summarized in Table 1. The output of the 3D LiDAR is a set of points, called a point cloud. The point cloud is a continuous terrain representation that is free of any artificial discretization. Hence, It is suitable for accurately computing the traversabilities at specific vehicle poses. Besides, it is a by-product of the SLAM module, so we do not spend additional computation time on building and maintaining terrain maps. Next, we will compute the vehicle pose and the traversability based on the point cloud.

### 3.2. Pose Estimation

A ground vehicle is always constrained to move on the surface of rough terrain, so the height, roll, and pitch of the vehicle are controlled by the local geometry of the terrain surface and need to be estimated. Formally, pose estimation can be defined as the following function:

(1) $$f_{\mathrm{pe}}:\;\left(P,\;\bar{s}\right)\overset{}{\to }\tilde{s}$$

where P is a point cloud that is constructed before trajectory planning. $\bar{s}={\left[\bar{x}\;\;\bar{y}\;\;{\bar{\theta }}_{z}\right]}^{T}\in$ SE(2) is a query pose, and $\tilde{s}={\left[\tilde{x}\;\;\tilde{y}\;\;\tilde{z}\;\;{\tilde{\theta }}_{x}\;\;{\tilde{\theta }}_{y}\;\;{\tilde{\theta }}_{z}\right]}^{T}\in$ SE(3) is the estimated pose on the terrain surface. Note that SE(2) is a state-space whose element is composed of *x*-coordinate, *y*-coordinate, and yaw. SE(3) is a state-space whose element is composed of *x*-coordinate, *y*-coordinate, *z*-coordinate, roll, pitch, and yaw. In the following, a pose estimation method developed assuming that the vehicle is static. This approach first calculates a roll and a pitch according to the wheel-terrain interaction, and then estimates the SE(3) pose of the vehicle based on its suspension system. The proposed pose estimation method is based on Point Clouds and vehicle Suspension models, so it is called PC-Sus.

#### 3.2.1. Euler Angle Estimation Based on Wheel-Terrain Interaction

First, the position of the vehicle’s wheels on the terrain surface is calculated. For a given query pose $\bar{s}={\left[\bar{x}\;\;\bar{y}\;\;{\bar{\theta }}_{z}\right]}^{T}$, the planar coordinates of the right front wheel are calculated as:

(2) $$\left[\begin{array}{c} x_{\mathrm{rf}}\\ y_{\mathrm{rf}} \end{array}\right]=\left[\begin{array}{cc} \cos{\bar{\theta }}_{z} & \sin{\bar{\theta }}_{z}\\ -\sin{\bar{\theta }}_{z} & \cos{\bar{\theta }}_{z} \end{array}\right]\left[\begin{array}{c} W^{\prime }\;/\;2\\ L^{\prime } \end{array}\right]+\left[\begin{array}{c} \bar{x}\\ \bar{y} \end{array}\right],$$

where $W^{\prime }$ and $L^{\prime }$ are the length of the vehicle’s axle and the wheelbase, respectively. Let $P_{\mathrm{rf}}$ denote the following 3D point set:

(3) $$P_{\mathrm{rf}}=\left\{p={\left[x\;\;y\;\;z\right]}^{T}\;|\;{\left(x-x_{\mathrm{rf}}\right)}^{2}+{\left(y-y_{\mathrm{rf}}\right)}^{2}<{\left(\frac{W_{\mathrm{tire}}}{2}\right)}^{2},\;p\in P\right\},$$

where $W_{\mathrm{tire}}$ is the width of the vehicle’s tire. $P_{\mathrm{rf}}$ is the set of the LiDAR points that are near the contact area between the vehicle’s tire and the terrain surface. Then, the center of gravity of the points in $P_{\mathrm{rf}}$ is computed as:

(4) $$p_{\mathrm{average}}=\frac{1}{n}\sum_{p\in P_{\mathrm{rf}}}p$$

where *n* is the number of points in $P_{\mathrm{rf}}$. Let $z_{\mathrm{rf}}$ be the *z*-coordinate of $p_{\mathrm{average}}$. Finally, the position of the right front wheel on the terrain surface can be represented by $p_{\mathrm{rf}}={\left[x_{\mathrm{rf}}\;\;y_{\mathrm{rf}}\;\;z_{\mathrm{rf}}\right]}^{T}$. The positions of the right back wheel, the left front wheel, and the left back wheel are represented by $p_{\mathrm{rb}}$, $p_{\mathrm{lf}}$, and $p_{\mathrm{lb}}$ respectively, which are all computed in a similar way.

In this paper, every three wheel positions are defined as a *triple*. For a given triple $\alpha ={\left[p_{1}\;\;p_{2}\;\;p_{3}\right]}^{T}$ ($p_{i}={\left[x_{i}\;\;y_{i}\;\;z_{i}\right]}^{T}$), the normal vector of the plane passing through these three points is:

(5) $$n\left(\alpha \right)=\left(p_{2}-p_{1}\right)\times \left(p_{3}-p_{1}\right)=\left|\begin{array}{ccc} i & j & k\\ x_{2}-x_{1} & y_{2}-y_{1} & z_{2}-z_{1}\\ x_{3}-x_{1} & y_{3}-y_{1} & z_{3}-z_{1} \end{array}\right|=ai+bj+ck={\left[a\;\;b\;\;c\right]}^{T},$$

where $i={\left[1\;\;0\;\;0\right]}^{T}$, $j={\left[0\;\;1\;\;0\right]}^{T}$, $k={\left[0\;\;0\;\;1\right]}^{T}$, $a=\left(y_{2}-y_{1}\right)\left(z_{3}-z_{1}\right)-\left(y_{3}-y_{1}\right)\left(z_{2}-z_{1}\right)$, $b=\left(z_{2}-z_{1}\right)\left(x_{3}-x_{1}\right)-\left(z_{3}-z_{1}\right)\left(x_{2}-x_{1}\right)$, and $c=\left(x_{2}-x_{1}\right)\left(y_{3}-y_{1}\right)-\left(x_{3}-x_{1}\right)\left(y_{2}-y_{1}\right)$. Then, the roll ($\theta_{x}\left(\alpha \right)$) and the pitch ($\theta_{y}\left(\alpha \right)$) of the plane that passes through the three points are calculated as:

(6) $$\theta_{x}\left(n(\alpha )\right)=\mathrm{sgn}\left(b\right)\arccos\frac{c}{\sqrt{b^{2}+c^{2}}}\;\;\;\;\;\;\;\;\;\;\theta_{y}\left(n(\alpha )\right)=\mathrm{sgn}\left(a\right)\arccos\sqrt{\frac{b^{2}+c^{2}}{a^{2}+b^{2}+c^{2}}}$$

where sgn is the sign function.

Recall that the wheel positions $p_{\mathrm{rf}}$, $p_{\mathrm{rb}}$, $p_{\mathrm{lf}}$ and $p_{\mathrm{lb}}$ have been computed previously. Let A be a set of triples: $\left\{{\left[p_{\mathrm{rf}}\;\;p_{\mathrm{lf}}\;\;p_{\mathrm{lb}}\right]}^{T},\;{\left[p_{\mathrm{rf}}\;\;p_{\mathrm{lf}}\;\;p_{\mathrm{rb}}\right]}^{T},\;{\left[p_{\mathrm{rf}}\;\;p_{\mathrm{lb}}\;\;p_{\mathrm{rb}}\right]}^{T},\;{\left[p_{\mathrm{lf}}\;\;p_{\mathrm{lb}}\;\;p_{\mathrm{rb}}\right]}^{T}\right\}$. Then, a roll ($\theta_{x}^{*}$) and a pitch ($\theta_{y}^{*}$) can be calculated as:

(7) $$\begin{array}{ll} \alpha_{x}^{*}=\underset{\alpha \in A}{\arg\max}|\theta_{x}\left(n(\alpha )\right)|\;\;\;\;\;\;\;\;\;\; & \alpha_{y}^{*}=\underset{\alpha \in A}{\arg\max}\left|\theta_{y}\left(n(\alpha )\right)\right|\\ \theta_{x}^{*}=\theta_{x}\left(n\left(\alpha_{x}^{*}\right)\right) & \theta_{y}^{*}=\theta_{y}\left(n\left(\alpha_{y}^{*}\right)\right) \end{array}$$

where $\alpha_{x}^{*}$ and $\alpha_{y}^{*}$ are the triples that make the roll and the pitch equal to the maximal absolute values, respectively. $\theta_{x}^{*}$ and $\theta_{y}^{*}$ are the maximal roll and pitch with signs (positive or negative), respectively. If the vehicle is assumed to be a rigid body without a suspension system, $\theta_{x}^{*}$ and $\theta_{y}^{*}$ will be the roll and the pitch of the vehicle, respectively.

#### 3.2.2. Pose Estimation Based on Suspension System

Note that $\theta_{x}^{*}$ and $\theta_{y}^{*}$ cannot be used as the roll and the pitch of the vehicle, because the above calculations do not consider the suspension model of the vehicle. The role of the suspension model is a theoretical basis used to compute the roll and the pitch. Without the suspension model, the whole vehicle can only be taken as a rigid body. However, a real vehicle is never a rigid body. Next, the roll and the pitch will be computed based on the suspension model. During the computation, an appropriate suspension model will be chosen based on $\theta_{x}^{*}$ to estimate the SE(3) pose ($\tilde{s}$) of the vehicle.

**Figure 4:**
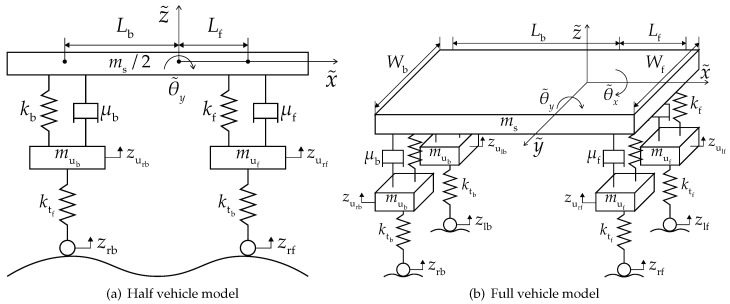
The models of vehicle suspension systems.

There are two well known passive suspension models: the half vehicle model and the full vehicle model, as shown in Figure 4a,b. Formally, the dynamic model of a half vehicle contains four linear differential equations [43]:

(8) $$\begin{array}{cc} m_{s}\ddot{\tilde{z}}/2 & =-\mu_{f}\left({\dot{z}}_{s_{\mathrm{rf}}}-{\dot{z}}_{u_{\mathrm{rf}}}\right)-\mu_{b}\left({\dot{z}}_{s_{\mathrm{rb}}}-{\dot{z}}_{u_{\mathrm{rb}}}\right)-k_{f}\left(z_{s_{\mathrm{rf}}}-z_{u_{\mathrm{rf}}}\right)-k_{b}\left(z_{s_{\mathrm{rb}}}-z_{u_{\mathrm{rb}}}\right)\\ I_{y}{\ddot{\tilde{\theta }}}_{y}/2 & =-\mu_{f}L_{f}\left({\dot{z}}_{s_{\mathrm{rf}}}-{\dot{z}}_{u_{\mathrm{rf}}}\right)+\mu_{b}L_{b}\left({\dot{z}}_{s_{\mathrm{rb}}}-{\dot{z}}_{u_{\mathrm{rb}}}\right)-k_{f}L_{f}\left(z_{s_{\mathrm{rf}}}-z_{u_{\mathrm{rf}}}\right)+k_{b}L_{b}\left(z_{s_{\mathrm{rb}}}-z_{u_{\mathrm{rb}}}\right)\\ m_{u_{f}}{\ddot{z}}_{u_{\mathrm{rf}}} & =\mu_{f}\left({\dot{z}}_{s_{\mathrm{rf}}}-{\dot{z}}_{u_{\mathrm{rf}}}\right)+k_{f}\left(z_{s_{\mathrm{rf}}}-z_{u_{\mathrm{rf}}}\right)+k_{t_{f}}\left(z_{\mathrm{rf}}-z_{u_{\mathrm{rf}}}\right)\\ m_{u_{b}}{\ddot{z}}_{u_{\mathrm{rb}}} & =\mu_{b}\left({\dot{z}}_{s_{\mathrm{rb}}}-{\dot{z}}_{u_{\mathrm{rb}}}\right)+k_{b}\left(z_{s_{\mathrm{rb}}}-z_{u_{\mathrm{rb}}}\right)+k_{t_{b}}\left(z_{\mathrm{rb}}-z_{u_{\mathrm{rb}}}\right) \end{array}$$

Note that the half vehicle model assumes that the roll of the vehicle is zero. The dynamic model of a full vehicle contains seven linear differential equations [44]:

(9) $$\begin{array}{cc} m_{s}\ddot{\tilde{z}}= & -\mu_{f}\left({\dot{z}}_{s_{\mathrm{rf}}}-{\dot{z}}_{u_{\mathrm{rf}}}\right)-\mu_{f}\left({\dot{z}}_{s_{\mathrm{lf}}}-{\dot{z}}_{u_{\mathrm{lf}}}\right)-\mu_{b}\left({\dot{z}}_{s_{\mathrm{rb}}}-{\dot{z}}_{u_{\mathrm{rb}}}\right)-\mu_{b}\left({\dot{z}}_{s_{\mathrm{lb}}}-{\dot{z}}_{u_{\mathrm{lb}}}\right)\\ \; & -k_{f}\left(z_{s_{\mathrm{rf}}}-z_{u_{\mathrm{rf}}}\right)-k_{f}\left(z_{s_{\mathrm{lf}}}-z_{u_{\mathrm{lf}}}\right)-k_{b}\left(z_{s_{\mathrm{rb}}}-z_{u_{\mathrm{rb}}}\right)-k_{b}\left(z_{s_{\mathrm{lb}}}-z_{u_{\mathrm{lb}}}\right)\\ I_{x}{\ddot{\tilde{\theta }}}_{x}= & -\mu_{f}W_{f}\left({\dot{z}}_{s_{\mathrm{rf}}}-{\dot{z}}_{u_{\mathrm{rf}}}\right)+\mu_{f}W_{f}\left({\dot{z}}_{s_{\mathrm{lf}}}-{\dot{z}}_{u_{\mathrm{lf}}}\right)-\mu_{b}W_{b}\left({\dot{z}}_{s_{\mathrm{rb}}}-{\dot{z}}_{u_{\mathrm{rb}}}\right)+\mu_{b}W_{b}\left({\dot{z}}_{s_{\mathrm{lb}}}-{\dot{z}}_{u_{\mathrm{lb}}}\right)\\ \; & -k_{f}W_{f}\left(z_{s_{\mathrm{rf}}}-z_{u_{\mathrm{rf}}}\right)+k_{f}W_{f}\left(z_{s_{\mathrm{lf}}}-z_{u_{\mathrm{lf}}}\right)-k_{b}W_{b}\left(z_{s_{\mathrm{rb}}}-z_{u_{\mathrm{rb}}}\right)+k_{b}W_{b}\left(z_{s_{\mathrm{lb}}}-z_{u_{\mathrm{lb}}}\right)\\ I_{y}{\ddot{\tilde{\theta }}}_{y}= & -\mu_{f}L_{f}\left({\dot{z}}_{s_{\mathrm{rf}}}-{\dot{z}}_{u_{\mathrm{rf}}}\right)-\mu_{f}L_{f}\left({\dot{z}}_{s_{\mathrm{lf}}}-{\dot{z}}_{u_{\mathrm{lf}}}\right)+\mu_{b}L_{b}\left({\dot{z}}_{s_{\mathrm{rb}}}-{\dot{z}}_{u_{\mathrm{rb}}}\right)+\mu_{b}L_{b}\left({\dot{z}}_{s_{\mathrm{lb}}}-{\dot{z}}_{u_{\mathrm{lb}}}\right)\\ \; & -k_{f}L_{f}\left(z_{s_{\mathrm{rf}}}-z_{u_{\mathrm{rf}}}\right)-k_{f}L_{f}\left(z_{s_{\mathrm{lf}}}-z_{u_{\mathrm{lf}}}\right)+k_{b}L_{b}\left(z_{s_{\mathrm{rb}}}-z_{u_{\mathrm{rb}}}\right)+k_{b}L_{b}\left(z_{s_{\mathrm{lb}}}-z_{u_{\mathrm{lb}}}\right)\\ m_{u_{f}}{\ddot{z}}_{u_{\mathrm{rf}}}= & \;\mu_{f}\left({\dot{z}}_{s_{\mathrm{rf}}}-{\dot{z}}_{u_{\mathrm{rf}}}\right)+k_{f}\left(z_{s_{\mathrm{rf}}}-z_{u_{\mathrm{rf}}}\right)+k_{t_{f}}\left(z_{\mathrm{rf}}-z_{u_{\mathrm{rf}}}\right)\\ m_{u_{f}}{\ddot{z}}_{u_{\mathrm{lf}}}= & \;\mu_{f}\left({\dot{z}}_{s_{\mathrm{lf}}}-{\dot{z}}_{u_{\mathrm{lf}}}\right)+k_{f}\left(z_{s_{\mathrm{lf}}}-z_{u_{\mathrm{lf}}}\right)+k_{t_{f}}\left(z_{\mathrm{lf}}-z_{u_{\mathrm{lf}}}\right)\\ m_{u_{b}}{\ddot{z}}_{u_{\mathrm{rb}}}= & \;\mu_{b}\left({\dot{z}}_{s_{\mathrm{rb}}}-{\dot{z}}_{u_{\mathrm{rb}}}\right)+k_{b}\left(z_{s_{\mathrm{rb}}}-z_{u_{\mathrm{rb}}}\right)+k_{t_{b}}\left(z_{\mathrm{rb}}-z_{u_{\mathrm{rb}}}\right)\\ m_{u_{b}}{\ddot{z}}_{u_{\mathrm{lb}}}= & \;\mu_{b}\left({\dot{z}}_{s_{\mathrm{lb}}}-{\dot{z}}_{u_{\mathrm{lb}}}\right)+k_{b}\left(z_{s_{\mathrm{lb}}}-z_{u_{\mathrm{lb}}}\right)+k_{t_{b}}\left(z_{\mathrm{lb}}-z_{u_{\mathrm{lb}}}\right) \end{array}$$

where $z_{s_{\mathrm{rf}}}=W_{f}{\tilde{\theta }}_{x}+L_{f}{\tilde{\theta }}_{y}+\tilde{z}$, $z_{s_{\mathrm{lf}}}=-W_{f}{\tilde{\theta }}_{x}+L_{f}{\tilde{\theta }}_{y}+\tilde{z}$, $z_{s_{\mathrm{rb}}}=W_{b}{\tilde{\theta }}_{x}-L_{b}{\tilde{\theta }}_{y}+\tilde{z}$, $z_{s_{\mathrm{lb}}}=-W_{b}{\tilde{\theta }}_{x}-L_{b}{\tilde{\theta }}_{y}+\tilde{z}$, and $z_{\mathrm{rf}}$, $z_{\mathrm{rb}}$, $z_{\mathrm{lf}}$, $z_{\mathrm{lb}}$ are the heights of the vehicle’s wheels on the terrain surface. The definitions of the constants in Equations (8) and (9) are shown in Table 2. The values of these constants are obtained by referring to the manufacturer’s specifications of the vehicle.

Note that during the generation of the initial path, the vehicle is assumed to be static and all the first-order and second-order derivatives in Equations (8) and (9) are equal to zero. Let $x={\left[\tilde{z}\;\;{\tilde{\theta }}_{x}\;\;{\tilde{\theta }}_{y}\;\;z_{u_{\mathrm{rf}}}\;\;z_{u_{\mathrm{lf}}}\;\;z_{u_{\mathrm{rb}}}\;\;z_{u_{\mathrm{lb}}}\right]}^{T}$ denote the *state vector* and $w={\left[z_{\mathrm{rf}}\;\;z_{\mathrm{rb}}\;\;z_{\mathrm{lf}}\;\;z_{\mathrm{lb}}\right]}^{T}$ denote the *disturbance vector*. Then, Equation (9) can be written as the following state-transition equation: 0=Ax+Ew, where A and E are fixed matrices called *state-transition matrix* and *disturbance matrix*, respectively. The solution of this equation is $x=-A^{-1}Ew$, which can be considered as a mapping $f_{\mathrm{full}}:\;{\left[z_{\mathrm{rf}}\;\;z_{\mathrm{rb}}\;\;z_{\mathrm{lf}}\;\;z_{\mathrm{lb}}\right]}^{T}\overset{}{\to }{\left[\tilde{z}\;\;{\tilde{\theta }}_{x}\;\;{\tilde{\theta }}_{y}\right]}^{T}$. For the half vehicle model, the mapping $f_{\mathrm{half}}:\;{\left[z_{\mathrm{rf}}\;\;z_{\mathrm{rb}}\right]}^{T}\overset{}{\to }{\left[\tilde{z}\;\;{\tilde{\theta }}_{y}\right]}^{T}$ can be obtained in a similar way. Finally, the SE(3) pose ($\tilde{s}$) of the vehicle is calculated as:

(10) $$\begin{array}{cc} \tilde{s}={\left[\tilde{x}\;\;\tilde{y}\;\;\tilde{z}\;\;{\tilde{\theta }}_{x}\;\;{\tilde{\theta }}_{y}\;\;{\tilde{\theta }}_{z}\right]}^{T}\;\;\;\;\;\mathrm{where} & \;\;\;\;\;{\left[\tilde{x}\;\;\tilde{y}\;\;{\tilde{\theta }}_{z}\right]}^{T}=\bar{s}={\left[\bar{x}\;\;\bar{y}\;\;{\bar{\theta }}_{z}\right]}^{T}\\ \mathrm{and} & \;\;\;\left\{\begin{array}{cc} {\left[\tilde{z}\;\;{\tilde{\theta }}_{y}\right]}^{T}=f_{\mathrm{half}}\left({\left[z_{\mathrm{rf}}\;\;z_{\mathrm{rb}}\right]}^{T}\right),\;{\tilde{\theta }}_{x}=\theta_{x}^{*}\; & \mathrm{if}\;|\theta_{x}^{*}|⩽\theta_{x}^{+},\\ {}[\tilde{z}\;\;{\tilde{\theta }}_{x}\;\;{\tilde{\theta }}_{y}{]}^{T}=f_{\mathrm{full}}\left({\left[z_{\mathrm{rf}}\;\;z_{\mathrm{rb}}\;\;z_{\mathrm{lf}}\;\;z_{\mathrm{lb}}\right]}^{T}\right)\; & \mathrm{otherwise}. \end{array}\right. \end{array}$$

If the roll is nearly zero, the pitch and the *z*-coordinate are computed using the half vehicle model. Otherwise, the SE(3) pose of the vehicle is computed using the full vehicle model. In summary, given the *z*-coordinates of the vehicle wheels, the half or full vehicle suspension model will become a linear system of equations with 4 or 7 unknown variables. Then, the roll and the pitch of the vehicle can be calculated via solving this linear system of equations.

### 3.3. Traversability Computation

**Figure 5:**
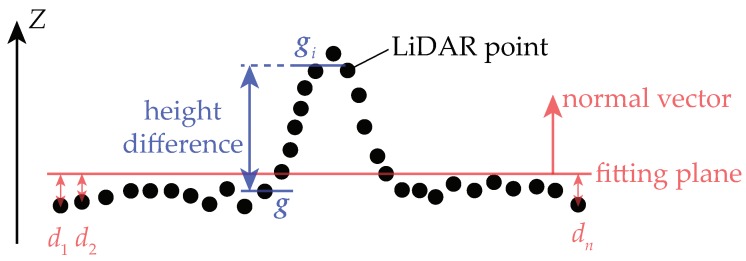
How are the terrain roughness and the height difference computed.

In addition to the roll and the pitch calculated by Equation (10), terrain roughness is also necessary in traversability computation. Let $P_{\mathrm{foot}}$ denote the set of all LiDAR points that are located in the footprint of the vehicle ($P_{\mathrm{foot}}\subset P$). Then, the center of gravity ($p_{\mathrm{foot}}$) of the points in $P_{\mathrm{foot}}$ and the associated covariance matrix are calculated as:

(11) $$p_{\mathrm{foot}}=\frac{1}{n}\sum_{p\in P_{\mathrm{foot}}}p$$

(12) $$\mathrm{cov}\left(p_{\mathrm{foot}},\;p_{\mathrm{foot}}\right)=\frac{1}{n}\sum_{p\in P_{\mathrm{foot}}}\left(p-p_{\mathrm{foot}}\right){\left(p-p_{\mathrm{foot}}\right)}^{T},$$

where *n* is the number of points in $P_{\mathrm{foot}}$. Figure 5 explains how the terrain roughness is computed. The terrain roughness (ρ) is defined as the residual of the fitting plane: $\sqrt{\sum_{j=1}^{n}d_{j}}$, where $d_{j}$ represents the distance between *i*th LiDAR point in $P_{\mathrm{foot}}$ and the fitting plane. Let $\lambda_{\min}$ be the minimum of the eigenvalues of $\mathrm{cov}(p_{\mathrm{foot}},\;p_{\mathrm{foot}})$. Then, the residual of the fitting plane is:

(13) $$\rho =\sqrt{\lambda_{\min}}$$

Besides, the height difference need to be computed. As shown in Figure 5, let g denote the grid in which the point ${\left[\tilde{x}\;\;\tilde{y}\right]}^{T}$ located, and let $P_{g}$ denote the set of all LiDAR points located in g ($P_{g}\subset P$). Then, the height of g can be calculated as:

(14) $$h_{g}=\frac{1}{n}\sum_{p\in P_{g}}p.z$$

where p.z is the *z*-coordinate of p. Let $g_{0},\;g_{1},\;\cdots ,\;g_{7}$ denote the eight-connected grids of g. Then, the height difference ($h_{d}$) is defined as: $|h_{g}-h_{g_{i}}|$, where $i=\lfloor \left[\left({\tilde{\theta }}_{z}+22.5\right)\;\mathrm{mod}\;360\right]/45\rfloor$ (${\tilde{\theta }}_{z}\in \left[0,\;360\right)$ and i∈[0,7]). The symbols “⌊⌋” and “mod” represent the round-down operator and the modulo operator, respectively. In fact, $g_{i}$ is the grid that is nearest to g in the heading direction of the vehicle.

Finally, the traversability is calculated as:

(15) $$\tau =\left\{\begin{array}{c} 0,\;\mathrm{if}\;|{\tilde{\theta }}_{x}|>{\tilde{\theta }}_{x\max}\;\mathrm{or}\;{\tilde{\theta }}_{y}<{\tilde{\theta }}_{y\min}\;\mathrm{or}\;{\tilde{\theta }}_{y}>{\tilde{\theta }}_{y\max}\;\mathrm{or}\;\rho >\rho_{\max}\;\mathrm{or}\;h_{d}>h_{\mathrm{dmax}}\\ 1-\left[w_{\tau_{1}}\max\left(\frac{{\tilde{\theta }}_{y}}{{\tilde{\theta }}_{y\min}},\;\frac{{\tilde{\theta }}_{y}}{{\tilde{\theta }}_{y\max}}\right)+w_{\tau_{2}}\frac{|{\tilde{\theta }}_{x}|}{{\tilde{\theta }}_{x\max}}+w_{\tau_{3}}\frac{\rho }{\rho_{\max}}+w_{\tau_{4}}\frac{h_{d}}{h_{\mathrm{dmax}}}\right],\;\mathrm{otherwise} \end{array}\right.$$

Note that the traversability (Equation (15)) is defined artificially. It is a relative value ranged from 0 to 1. In this work, the definition is that if one of the roll (${\tilde{\theta }}_{x}$), pitch (${\tilde{\theta }}_{y}$), terrain roughness (ρ), or height difference ($h_{d}$) exceeds the respective limit value, the traversability is 0. Otherwise, the traversability is the weighted sum of them, normalized by their respective limit values. Note that a vehicle is usually not front-back symmetrical, so there are two different limit values (${\tilde{\theta }}_{y\min}$ and ${\tilde{\theta }}_{y\max}$) for the pitch.

## 4. Trajectory Planning

**Figure 6:**
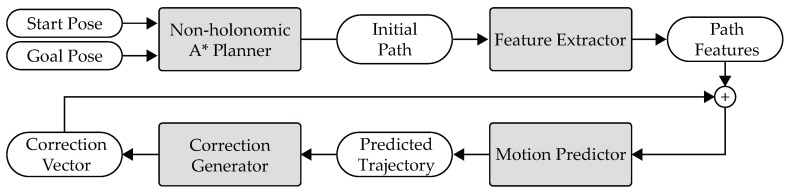
Overview of the trajectory planning approach.

In this section, an initial path will first be generated, which is subject to the non-holonomic constraints of a car-like vehicle. Then, this path will be converted into a trajectory, which will be optimized in terms of safety, traversability, time consumption, trajectory smoothness, and so on. Note that the proposed planner and optimizer are called on demand, so the frequency of planning and optimization is not fixed. For example, if the solution trajectory is blocked by a new sensed obstacle, then the planner and optimizer will be called to generate a new trajectory. The overview of this section is shown in Figure 6. The proposed trajectory planner is based on Terrain Shapes and vehicle Suspension models, so it is called TS-Sus.

### 4.1. Non-Holonomic A*

For a given planning query (a start pose and a goal pose in the SE(2) space), an initial path is first found by a non-holonomic A* planner, which takes into account the non-holonomic constraints, the proximity to an obstacle, and the traversability. Next, the non-holonomic constraints of a car-like vehicle will be introduced, and then the node expansion and the cost function of the non-holonomic A* will be described.

#### 4.1.1. Non-Holonomic Constraints

**Figure 7:**
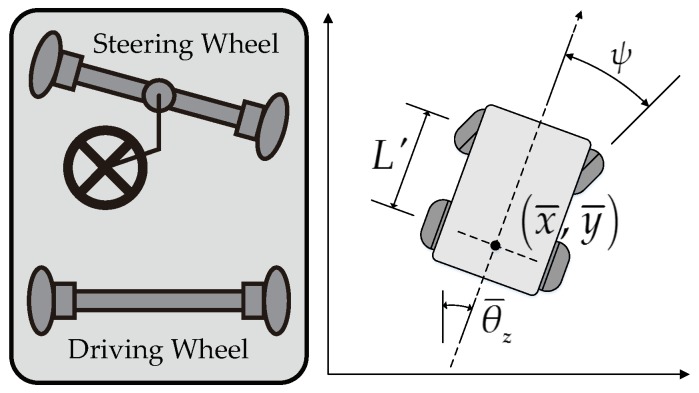
A car-like vehicle and its kinematic model.

For a mechanical system, kinematic constraints are described by the relations between the position and the velocity of the system. The kinematic constraints that cannot be integrated to the form containing only the position are called non-holonomic constraints. A system subject to non-holonomic constraints is called a non-holonomic system. A car-like vehicle is a non-holonomic system. It cannot move sideways, and its turning radius is lower bounded. Formally, the non-holonomic constraints that a car-like vehicle (shown in Figure 7) satisfies are written as:

(16) $$\left[\begin{array}{c} \dot{\bar{x}}\\ \dot{\bar{y}}\\ {\dot{\bar{\theta }}}_{z} \end{array}\right]=\left[\begin{array}{c} v\cdot \sin{\bar{\theta }}_{z}\\ v\cdot \cos{\bar{\theta }}_{z}\\ \left(v\cdot \tan\psi \right)\;/\;L^{\prime } \end{array}\right],$$

where ${\left[\bar{x}\;\;\bar{y}\;\;{\bar{\theta }}_{z}\right]}^{T}$ is the SE(2) pose of the vehicle, and *v* is the longitudinal velocity of the vehicle. ψ is the steering angle, and $L^{\prime }$ is the wheelbase.

#### 4.1.2. Node Expansion

**Figure 8:**
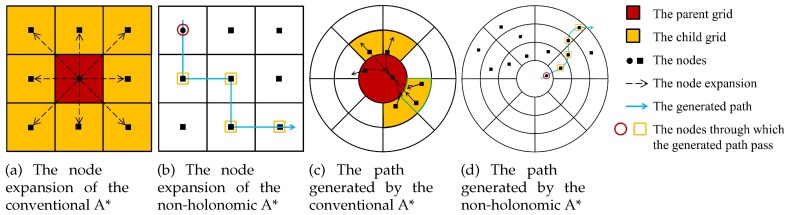
The differences between the conventional A* and the non-holonomic A*.

In this section, a non-holonomic A* search is performed to find a path satisfying the non-holonomic constraints. Figure 8 shows the differences between the conventional A* and the non-holonomic A*. On the one hand, the conventional A* treats a car-like vehicle as a point without orientation and only visits grid centers, as shown in Figure 8a. As a result, the path generated by the conventional A* is piecewise-linear, which is a sequence of vehicle positions ${\left[{\bar{x}}_{i}\;\;{\bar{y}}_{i}\right]}^{T},\;i=1,2,\cdots ,n$ (as shown Figure 8b). On the other hand, the non-holonomic A* considers a car-like vehicle as a vector. The child poses are generated by assuming some different steering angles and a fixed forward/reverse velocity in Equation (16). This method associates vehicle poses with grids, so any continuous pose of the vehicle can be visited. Therefore, the resultant path satisfies the non-holonomic constraints, as shown in Figure 8b. Mathematically, the path generated by the non-holonomic A* is a sequence of SE(2) poses ${\bar{s}}_{i}={\left[{\bar{x}}_{i}\;\;{\bar{y}}_{i}\;\;{\bar{\theta }}_{z_{i}}\right]}^{T},\;i=1,2,\cdots ,n$, which can be mapped to SE(3) poses using the pose estimation method introduced in Section 3.2.

The shapes of the grids in the conventional A* and the non-holonomic A* are also different. The conventional A* planner and the non-holonomic A* planner search square-shaped grids and sector-shaped grids, respectively. The sector-shaped grids can well represent the characteristics of point cloud data (high resolution for the LiDAR points near the vehicle, and low resolution for the points that are far from the vehicle).

#### 4.1.3. Cost Function

The order of the node expansions in the non-holonomic A* is partly determined by *movement costs*. There are different costs for different types of movements. For example, moving on the uneven ground leads to a greater cost than moving on flat ground, and the cost of moving reverse is greater than that of moving forward. In this work, the cost ($f_{m}$) of moving from a parent pose (${\tilde{s}}_{i}$∈ SE(3)) to a child pose (${\tilde{s}}_{i+1}$∈ SE(3)) is computed as:

(17) $$\begin{array}{cc} f_{m}\left({\tilde{s}}_{i+1},\;{\tilde{s}}_{i}\right)= & \;w_{m_{1}}\epsilon_{\mathrm{reverse}}\frac{\mathrm{length}({\tilde{s}}_{i+1},\;{\tilde{s}}_{i})}{N_{m_{1}}}+w_{m_{2}}\frac{\epsilon_{\mathrm{switch}}}{N_{m_{2}}}\\ + & \;w_{m_{3}}\frac{\max(0,\;d_{\mathrm{omax}}-d_{o}^{i+1})}{N_{m_{3}}}+w_{m_{4}}\frac{1-\tau_{i+1}}{N_{m_{4}}} \end{array},\;d_{o}^{i+1}\neq 0\;\mathrm{and}\;\tau_{i+1}\neq 0\;\mathrm{and}\;∥{\tilde{s}}_{i+1}-{\tilde{s}}_{i}∥⩽\xi$$

where $w_{m_{i}}$ (i=1,2,3,4) is a weight factor (determined empirically) that indicates the importance of the respective cost, and $N_{m_{i}}$ (i=1,2,3,4) is a normalization factor that is determined as the largest value of the respective cost. $d_{o}^{i+1}$ is the distance between ${\tilde{s}}_{i+1}$ and the obstacle nearest to ${\tilde{s}}_{i+1}$. $d_{\mathrm{omax}}$, called *safe distance*, is determined according to the vehicle size and the environment. $\tau_{i+1}$ is the traversability of ${\tilde{s}}_{i+1}$. ξ is the resolution of the path generated by the non-holonomic A* planner. Besides, $\epsilon_{\mathrm{reverse}}$ and $\epsilon_{\mathrm{switch}}$ are computed as:

(18) $$\epsilon_{\mathrm{reverse}}=1+\delta \left(1+\mathrm{sgn}(v_{i+1})\right)P_{\mathrm{reverse}}\;\epsilon_{\mathrm{switch}}=\delta \left(2-\left|\mathrm{sgn}\left(v_{i+1}\right)-\mathrm{sgn}\left(v_{i}\right)\right|\right)P_{\mathrm{switch}}$$

where

(19) $$\delta \left(u\right)=\left\{\begin{array}{cc} 0, & u\neq 0\\ 1, & u=0 \end{array}\right.\;\mathrm{sgn}\left(v\right)=\left\{\begin{array}{cc} -1, & v<0\\ 0, & v=0\\ 1, & v>0 \end{array}\right.$$

and $P_{\mathrm{reverse}}$, $P_{\mathrm{switch}}$ are the multiplicative penalty applied to driving reverse and the additive penalty applied to switching direction, respectively. Finally, $\mathrm{length}({\tilde{s}}_{i+1},\;{\tilde{s}}_{i})$ is the length of the path segment connecting the parent pose (${\tilde{s}}_{i}$) and the child pose (${\tilde{s}}_{i+1}$). It can be calculated as:

(20) $$\mathrm{length}\left({\tilde{s}}_{i+1},\;{\tilde{s}}_{i}\right)=\left\{\begin{array}{cc} \Delta {\tilde{\theta }}_{z}r_{\mathrm{turn}},\; & \Delta {\tilde{\theta }}_{z}\neq 0\\ {\left({\Delta \tilde{x}}^{2}+{\Delta \tilde{y}}^{2}+{\Delta \tilde{z}}^{2}\right)}^{1/2},\; & \Delta {\tilde{\theta }}_{z}=0 \end{array}\right.$$

where $r_{\mathrm{turn}}$ is the maximal turning radius of the vehicle. $\Delta \tilde{x}$ is computed as ${\tilde{x}}_{i+1}-{\tilde{x}}_{i}$. $\Delta \tilde{y}$, $\Delta \tilde{z}$, and $\Delta {\tilde{\theta }}_{z}$ are all computed in a similar way. During the non-holonomic A* search, the cost ($f_{g}$, called *cost-to-come*) of moving from the start pose (${\tilde{s}}_{0}$) to the current pose (${\tilde{s}}_{i}$) can be calculated as:

(21) $$f_{g}\left({\tilde{s}}_{i}\right)=f_{m}\left({\tilde{s}}_{i},\;{\tilde{s}}_{i-1}\right)+f_{g}\left({\tilde{s}}_{i-1}\right)=f_{m}\left({\tilde{s}}_{i},\;{\tilde{s}}_{i-1}\right)+f_{m}\left({\tilde{s}}_{i-1},\;{\tilde{s}}_{i-2}\right)+\cdots +f_{m}\left({\tilde{s}}_{1},\;{\tilde{s}}_{0}\right).$$

Note that the pose whose $d_{o}$ or τ equals 0 is not considered in Equation (17), because it is the non-traversable pose that is not expanded by the A* search. The computational complexity of A* is well known: $O(b^{d})$, where *b* is the branching factor of the A* search tree, and *d* is the depth of the goal node.

### 4.2. Trajectory Optimization

The non-holonomic A* only accounts for the kinematic model of the ground vehicle. However, the pose of the vehicle is also largely affected by its suspension model in a non-planar environment. Besides, the initial path produced by the non-holonomic A* is usually not smooth enough and worthy of further improvement. Next, the features of the initial path will first be extracted and the trajectory of the vehicle will be predicted. This prediction is based on the extracted features and the suspension model of the vehicle. Then, the predicted trajectory corresponding to the features will be optimized in terms of smoothness, traversability, and so on.

#### 4.2.1. Feature Extraction

**Figure 9:**
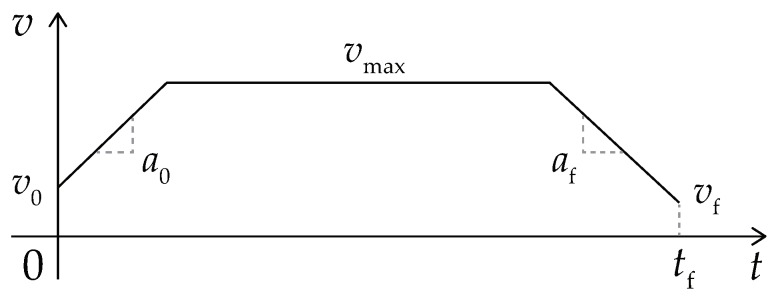
The trapezoidal profile of the longitudinal velocity.

**Figure 10:**
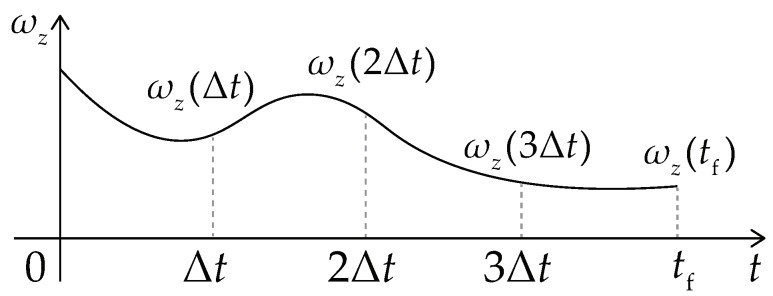
The spline profile of the angular velocity.

The initial path is a sequence of SE(3) poses, which is a high-dimensional vector and hard to be optimized directly. So the features of this path are first extracted to descend its dimension. The feature vector (u) is defined as:

(22) $$u={\left[u_{v}^{T}\;\;u_{\_z}^{T}\right]}^{T},\;u_{v}={\left[v_{0}\;\;a_{0}\;\;v_{\max}\;\;a_{f}\;\;v_{f}\;\;t_{f}\right]}^{T}\;\mathrm{and}\;u_{\omega_{z}}={\left[\omega_{z}\left(0\right)\;\;\omega_{z}\left(\Delta t\right)\;\;\omega_{z}\left(2\Delta t\right)\;\;\cdots \;\;\omega_{z}\left(t_{f}\right)\right]}^{T}$$

where $u_{v}$ determines the parameters of the trapezoid shown in Figure 9, and $u_{\omega_{z}}$ determines the knot-points of the spline curve shown in Figure 10. $v_{0}$, $a_{0}$, $v_{\max}$, $a_{f}$, and $v_{f}$ are the start velocity, the start acceleration, the maximal velocity (or called the traverse velocity), the terminal acceleration, and the terminal velocity. $\omega_{z}\left(t\right)$ is the angular velocity of the vehicle at time instant *t*.

The initial path produced by the non-holonomic A* contains no information about the velocity and the acceleration of the vehicle, so $v_{0}$, $a_{0}$, $v_{\max}$, $a_{f}$ and $v_{f}$ are set manually according to the limitation of the vehicle. $t_{f}$ is the time spent on moving from the start pose to the goal pose, and it can be calculated as:

(23) $$t_{f}=\left[l_{f}+\frac{{\left(v_{\max}-v_{0}\right)}^{2}}{2a_{0}}-\frac{{\left(v_{\max}-v_{f}\right)}^{2}}{2a_{f}}\right]\;/\;v_{\max}$$

where $l_{f}$ is the length of the initial path. Once $u_{v}$ is known, the mapping from the time instant (*t*) to the traveled distance of the vehicle (*l*) can be obtained via integration. Besides, for a given initial path, the mapping from *l* to the yaw of the vehicle (${\tilde{\theta }}_{z}$) can be obtained via interpolation. Finally, the mapping from *t* to ${\tilde{\theta }}_{z}$ ($t\in [0,\;t_{f}]$) can be obtained. Let *K* be the dimension of $u_{\omega_{z}}$ and $\Delta t=t_{f}/\left(K-1\right)$. Then, $u_{\omega_{z}}$ can be calculated approximately using forward differences:

(24) $$\omega_{z}\left(t\right)=\frac{d{\tilde{\theta }}_{z}}{dt}\approx \frac{{\tilde{\theta }}_{z}\left(t+e\right)-{\tilde{\theta }}_{z}\left(t\right)}{e}$$

#### 4.2.2. Motion Prediction

Before the feature vector is optimized, it should first be mapped to a vehicle trajectory by a motion prediction method, which contains two steps: motion simulation and pose estimation. Note that in the motion prediction the vehicle is not assumed to be static. This means that the derivatives in Equation (9) can be non-zero, so the pose estimation in the motion prediction is different from that in Section 3.2.

The motion simulation works as follows. Given the SE(3) pose of the vehicle at the current time instant $t_{i}$ ($t_{i}\in \left[0,\;t_{f}\right]$) and a feature vector (u), Euler’s method is used to calculate the planar position and the yaw at the next time instant $t_{i+1}$. This calculation is based on the following vehicle motion model:

(25) $$\left[\begin{array}{c} \dot{\tilde{x}}\\ \dot{\tilde{y}}\\ {\dot{\tilde{\theta }}}_{z} \end{array}\right]=\left[\begin{array}{ccc} \cos{\tilde{\theta }}_{z}\cos{\tilde{\theta }}_{y} & \cos{\tilde{\theta }}_{z}\sin{\tilde{\theta }}_{y}\sin{\tilde{\theta }}_{x}-\sin{\tilde{\theta }}_{z}\cos{\tilde{\theta }}_{x} & 0\\ \sin{\tilde{\theta }}_{z}\cos{\tilde{\theta }}_{y} & \sin{\tilde{\theta }}_{z}\sin{\tilde{\theta }}_{y}\sin{\tilde{\theta }}_{x}+\cos{\tilde{\theta }}_{z}\cos{\tilde{\theta }}_{x} & 0\\ 0 & 0 & \frac{\cos{\tilde{\theta }}_{x}}{\cos{\tilde{\theta }}_{y}} \end{array}\right]\left[\begin{array}{c} v\\ 0\\ \omega_{z} \end{array}\right].$$

The pose estimation works as follows. Given the planar position and the yaw at $t_{i+1}$, Equations (2)–(4) are used to compute the heights of the four vehicle wheels ($z_{\mathrm{rf}}$, $z_{\mathrm{rb}}$, $z_{\mathrm{lf}}$, $z_{\mathrm{lb}}$), which are the disturbance inputs of the vehicle’s suspension model (shown in Equation (9)). Then, Euler’s method is used again to compute the height ($\tilde{z}$), the roll (${\tilde{\theta }}_{x}$), and the pitch (${\tilde{\theta }}_{y}$) of the vehicle at $t_{i+1}$ based on Equation (9). A vehicle trajectory, which is a sequence of SE(3) vehicle poses, is generated when $t_{i}$ is equal to $t_{f}$. Note that this vehicle trajectory is different from the initial path produced by the non-holonomic A*. The poses along the trajectory are all time-indexed, and the trajectory is generated without assuming that the vehicle is static.

#### 4.2.3. Numerical Optimization

To generate an optimal trajectory, the optimizer must adjust the feature vector (u) to satisfy the constraint: $\Delta {\tilde{s}}_{f}\left(u\right)={\tilde{s}}_{f}-\tilde{s}\left(t_{f}\right)=0$, and minimize a cost function ($f_{t}\left(u\right)$), where ${\tilde{s}}_{f}$ is the goal pose and $\tilde{s}\left(t_{f}\right)$ is the terminal pose of the trajectory generated by the motion prediction. This is a constrained optimization problem, which can be solved using the *method of Lagrange multipliers*. The basic idea is to convert a constrained problem into a form such that the derivative test of an unconstrained problem can be applied. The Lagrange function (L) is written as:

(26) $$L\left(u,\;\lambda \right)=f_{t}\left(u\right)+\lambda^{T}\Delta {\tilde{s}}_{f}\left(u\right),$$

where λ is the Lagrange multiplier vector. The necessary conditions for optimality are:

(27) $$\nabla_{u,\lambda }L\left(u,\;\lambda \right)=0\;\;\;⟺\;\;\;\left\{\begin{array}{c} \frac{\partial L(u,\;\lambda )}{\partial u}=\frac{\partial f_{t}\left(u\right)}{\partial u}+\lambda^{T}\frac{\partial \Delta {\tilde{s}}_{f}\left(u\right)}{\partial u}=0^{T}\\ \frac{\partial L(u,\;\lambda )}{\partial \lambda }=\Delta {\tilde{s}}_{f}\left(u\right)=0 \end{array}\right..$$

Equation (27) can be solved using Newton’s method. The feature vector and the Lagrange multiplier vector are adjusted iteratively until a sufficiently precise value is reached. According to Newton’s method, a correction vector for u and λ at each iteration of the optimization is computed as:

(28) $$\left[\begin{array}{c} \Delta u\\ \Delta \lambda \end{array}\right]=-\beta H^{-1}J=-\beta {\left[\begin{array}{cc} \frac{\partial^{2}L(u,\;\lambda )}{{\partial u}^{2}} & {\frac{\partial \Delta {\tilde{s}}_{f}\left(u\right)}{\partial u}}^{T}\\ \frac{\partial \Delta {\tilde{s}}_{f}\left(u\right)}{\partial u} & 0 \end{array}\right]}^{-1}\left[\begin{array}{c} {\frac{\partial L(u,\;\lambda )}{\partial u}}^{T}\\ \Delta {\tilde{s}}_{f}\left(u\right) \end{array}\right],$$

where H and J are the Hessian matrix and the Jacobian matrix of the Lagrange function. A step size scaling factor (β) is used to improve numerical stability. In the implementation, forward differences are used to estimate the partial derivatives:

(29) $$\frac{\partial \Delta {\tilde{s}}_{i}\left(u\right)}{\partial u_{j}}\approx \frac{\Delta {\tilde{s}}_{i}\left(u_{1},\;u_{2},\;\cdots ,\;u_{j}+e,\;\cdots ,\;u_{n}\right)-\Delta {\tilde{s}}_{i}\left(u\right)}{e}$$

(30) $$\begin{array}{cc} \frac{\partial^{2}L(u)}{\partial u_{k}\partial u_{l}}\approx \frac{\Delta^{2}L}{e^{2}},\;\Delta^{2}L= & \;L(u_{1},\;u_{2},\;\cdots ,\;u_{k}+e,\;\cdots ,\;u_{l}+e,\;\cdots ,\;u_{n})\\ - & \;L(u_{1},\;u_{2},\;\cdots ,\;u_{k}+e,\;\cdots ,\;u_{n})\\ - & \;L\left(u_{1},\;u_{2},\;\cdots ,\;u_{l}+e,\;\cdots ,\;u_{n}\right)+L\left(u\right). \end{array}$$

where $\Delta {\tilde{s}}_{i}$ is the *i*th element of $\Delta {\tilde{s}}_{f}$, and $u_{j}$ is the *j*th element of u.

Note that the cost function ($f_{t}\left(u\right)$) used in the trajectory optimization is different from that used in the non-holonomic A*. The former is used to compute the cost of a trajectory corresponding to u. The poses along a trajectory are time-indexed, so $f_{t}\left(u\right)$ often accounts for more optimization criteria such as velocity, acceleration and time consumption. Next, the optimization criteria that are considered by $f_{t}\left(u\right)$ will be introduced.

Firstly, the proximity ($d_{o}$) of the trajectory to an obstacle and the traversability (τ) of the trajectory are included. The cost functions corresponding to them are written as:

(31) $$f_{d_{o}}\left(u\right)f_{d_{o}}\left(f_{\mathrm{mp}}\left(u\right)\right)=f_{d_{o}}\left(t\right)=\frac{1}{n}\sum_{i=1}^{n}\max\left(0,\;d_{\mathrm{omax}}-d_{o}^{i}\right)\;f_{\tau }\left(u\right)=\frac{1}{n}\sum_{i=1}^{n}1-\tau_{i}$$

where $f_{\mathrm{mp}}$: $u\overset{}{\to }t$ is the motion prediction, and t is a vehicle trajectory. *n* is the number of poses along t, and the other notations are the same as the notations in Equation (17). For clarity, t will always be replaced by u in the following definitions of cost functions. That is to say, if a cost is calculated based on the properties of a vehicle trajectory, the feature vector (u) will always be mapped to a trajectory (t) based on $f_{\mathrm{mp}}$.

Secondly, the velocity and the acceleration should be bounded from above and below according to the manufacturer’s specifications of the vehicle. For example, the cost function ($f_{v}$) corresponding to the longitudinal velocity is defined as:

(32) $$f_{v}\left(u\right)=\frac{1}{n}\sum_{i=1}^{n}\max\left(0,\;v_{\lim}^{-}-v_{i},\;v_{i}-v_{\lim}^{+}\right),\;v_{\lim}^{-}<0\;\;\mathrm{and}\;\;v_{\lim}^{+}>0,$$

where $v_{i}$ is the longitudinal velocity of the vehicle when it is at the *i*th pose along the trajectory. $v_{\lim}^{-}$ and $v_{\lim}^{+}$ are the reversing velocity limit and the forward velocity limit of the vehicle, respectively. In a similar way, the cost functions corresponding to the angular velocity, the longitudinal acceleration and the angular acceleration of the vehicle are defined as $f_{\omega_{z}}\left(u\right)$, $f_{a}\left(u\right)$, and $f_{\alpha_{z}}\left(u\right)$, respectively.

Thirdly, the time consumption ($t_{f}$) and the length ($l_{f}$) of the trajectory should also be included. The cost functions corresponding to them are $f_{t_{f}}\left(u\right)=t_{f}$ and $f_{l_{f}}\left(u\right)=l_{f}$. $l_{f}$ is computed by Equation (23) when $t_{f}$ is known. Fourthly, the trajectory should be optimized in terms of smoothness to reduce unnatural swerves. The cost function ($f_{\sigma }$) corresponding to the trajectory smoothness is defined as:

(33) $$f_{\sigma }\left(u\right)=\frac{1}{n-2}\sum_{i=2}^{n-1}{\left({\left[{\tilde{x}}_{i+1}\;\;{\tilde{y}}_{i+1}\;\;{\tilde{z}}_{i+1}\right]}^{T}-2{\left[{\tilde{x}}_{i}\;\;{\tilde{y}}_{i}\;\;{\tilde{z}}_{i}\right]}^{T}+{\left[{\tilde{x}}_{i-1}\;\;{\tilde{y}}_{i-1}\;\;{\tilde{z}}_{i-1}\right]}^{T}\right)}^{2}.$$

Note that the *z*-coordinate is also considered in the calculation of the smoothness cost. In this way, a trajectory that prevents the vehicle from rising and falling frequently can be obtained. Finally, the total cost function ($f_{t}\left(u\right)$) is computed as a weighted sum of all the above-mentioned cost functions.

The computational complexity of the trajectory optimization is O(mn), where *m* is the number of optimization iteration, and *n* is the number of poses along the trajectory that is optimized. Recall that the computational complexity of A* is $O(b^{d})$, so the computational complexity of the proposed approach is $O(b^{d}+mn)$.

## 5. Experimental Results and Discussion

This section provides a precise description of the experimental results and discusses them. Moreover, the proposed traversability assessor and trajectory planner are analyzed according to these results. The experiments were conducted in both the simulation and the real world, with the same set of parameter values. The half and full vehicle suspension models used in experiments are shown in Figure 4, Equations (8) and (9). The programs of the proposed approaches are executed on a single core of a 3.2 GHz Intel Core i5-3470 processor.

### 5.1. Simulation Experiments

In the simulation experiments, virtual terrain surfaces were created using a terrain editor called *EarthSculptor*, and the point clouds of these virtual terrain surfaces were generated by a virtual HDL-64E S2 LiDAR in a simulator called Gazebo, which was run on the Robot Operating System (ROS). Besides, a virtual vehicle with the samespecifications as our real vehicle (an all-terrain vehicle) was created in the Gazebo simulator.

**Figure 11:**
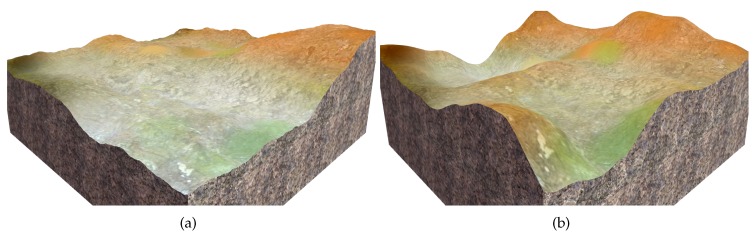
The virtual terrain surfaces used to test the proposed pose estimation method.

Let S denote the following set: $\left\{{\left[\bar{x}\;\;\bar{y}\;\;{\bar{\theta }}_{z}\right]}^{T}\;|\;\bar{x}/1\in Z,\;\bar{y}/1\in Z,\;{\bar{\theta }}_{z}/0.1\in Z,\;\bar{x}\in \left[0,\;W_{m}\right],\;\bar{y}\in \left[0,\;L_{m}\right],\;{\bar{\theta }}_{z}\in \left[0,\;2\pi \right)\right\}$, where $W_{m}$ and $L_{m}$ are the width and the length of a terrain surface (in meters), respectively. Z is the set of all integers. For each terrain surface in Figure 11, all the query poses in S were mapped to SE(3) poses using the proposed pose estimation method. Then, to acquire the ground truths of the vehicle’s height, roll, and pitch, the virtual vehicle was launched at each query pose in S, and the corresponding SE(3) pose was acquired via the ROS message published by the Gazebo simulator. Finally, the errors of our pose estimation method (PC-Sus) could be calculated. Similarly, we computed the errors of a pose estimation method [2] (called DEM-Kin) that is based on Digital Elevation Maps and vehicle Kinematic models, and another pose estimation method [45] (called Kin-GP-VE) that is based on Kinematics, Gaussian Processes, and Vehicle Experiences. Table 3 shows these errors and the improvement in pose estimation using the proposed PC-Sus method. The Root Mean Squared Errors (RMSEs) in roll and pitch estimation were reduced by approximately 35% and 47% over the Kin-GP-VE method and the DEM-Kin method, respectively.

**Figure 12:**
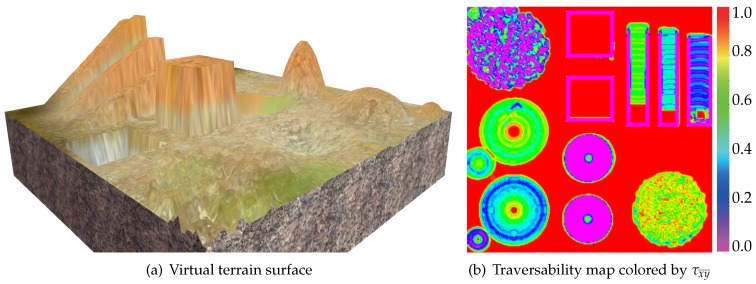
A virtual terrain surface and its traversability map.

Let Θ denote the following set: $\left\{{\bar{\theta }}_{z}\;|\;{\bar{\theta }}_{z}/0.1\in Z,\;{\bar{\theta }}_{z}\in \left[0,\;2\pi \right)\right\}$. Then, the traversability at ${\left[\bar{x}\;\;\bar{y}\right]}^{T}$ can be defined as:

(34) $$\tau_{\bar{x}\bar{y}}\max_{{\bar{\theta }}_{z}\in \Theta }\;\tau \left(f_{\mathrm{pe}}\left({\left[\bar{x}\;\;\bar{y}\;\;{\bar{\theta }}_{z}\right]}^{T}\right)\right),$$

where τ is calculated using Equation (15), and $f_{\mathrm{pe}}$ is shown in Equation (1). Figure 12 shows the heat map colored by $\tau_{\bar{x}\bar{y}}$, which is called traversability map. According to the results, the proposed method can evaluate the traversabilities of different terrain shapes, such as ramps, pits, and so on. Note that the traversability maps were constructed only for the purpose of validating the proposed traversability assessor. The traversability map would not be built in trajectory planning. During the process of planning, only the traversabilities of the query poses would be computed.

**Figure 13:**
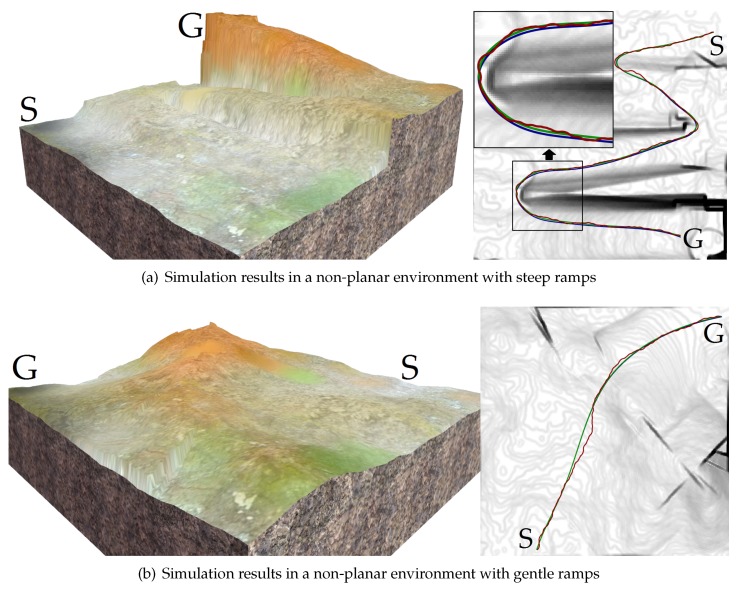
The initial paths (red), the trajectories (green) after optimization, and the real trajectories (blue) of the vehicle on the virtual terrain surfaces. For clarity, the trajectories are drawn on the grayscale traversability maps.

**Figure 14:**
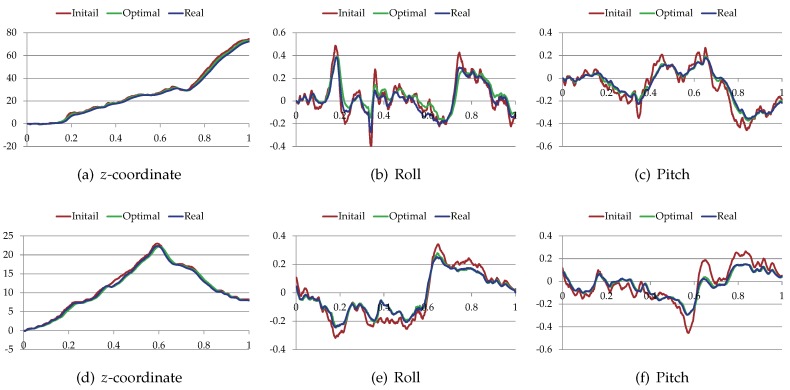
The *z*-coordinate, the roll and the pitch of the vehicle along the paths and the trajectories. (**a**–**c**) The vehicle state along the trajectories shown in Figure 13a; (**d**–**f**) The vehicle state along the trajectories shown in Figure 13b.

The proposed trajectory planner was tested on different terrain surfaces. Figure 13 shows the initial paths (red) produced by the non-holonomic A*, the trajectories (green) after optimization, and the real trajectories (blue) of the virtual vehicle. The blue trajectories were generated by making the virtual vehicle track the green trajectories. For clarity, all the above trajectories were drawn on the grayscale traversability maps of the terrain surfaces. Figure 14 shows the *z*-coordinate, the roll and the pitch of the vehicle along these paths and trajectories. Table 4 shows the performance comparison of these paths and trajectories. Note that $d_{o}$ is +∞ when there is no obstacle in the environments. According to the results, the initial paths had many unnatural swerves (according to Figure 13). Although such paths were drivable, they often led to excessive steering of the vehicle. Compared with the initial paths, the trajectories after optimization were shorter, smoother and had higher traversabilities (according to Figure 14 and Table 4). Therefore, the total cost of the trajectory is reduced after the optimization.

**Figure 15:**
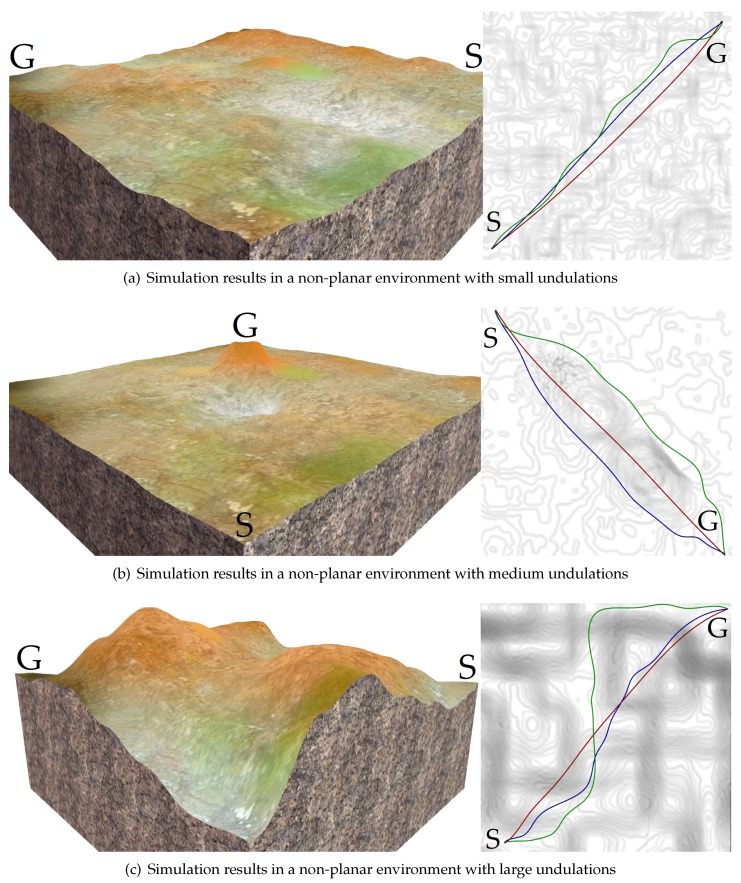
The trajectories generated by NoTS (red), TS-NoSus (green), and TS-Sus (blue).

**Figure 16:**
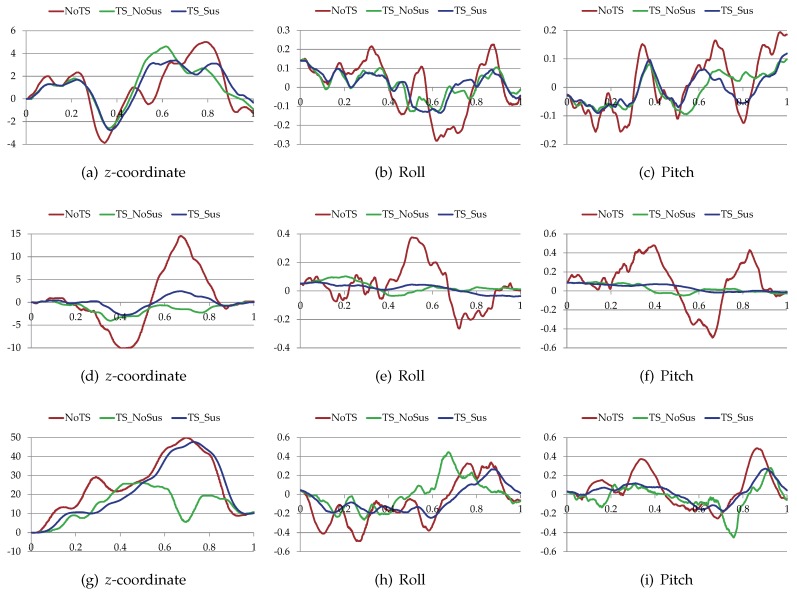
The *z*-coordinate, the roll and the pitch of the vehicle along the trajectories. (**a**–**c**) The vehicle state along the trajectories shown in Figure 15a; (**d**–**f**) The vehicle state along the trajectories shown in Figure 15b; (**g**–**i**) The vehicle state along the trajectories shown in Figure 15c.

Furthermore, Figure 15, Figure 16, and Table 5 compare the proposed trajectory planner (TS-Sus) with two other state-of-the-art trajectory planners. One [46] (referred to as NoTS) of the two methods does Not account for the Terrain Shape. The other one [26] (referred to as TS-NoSus) takes the Terrain Shape into consideration, but it ignores the Suspension system of the vehicle. In the environment shown in Figure 15a, the TS-NoSus method computed a trajectory that avoided the small terrain undulations. This trajectory was highly traversable (according Figure 16a–c and Table 5) but not smooth (according to Table 5). In fact, the all-terrain vehicle could directly pass through the small terrain undulations without reducing the traversability, due to the shock absorption of its suspension system. The proposed TS-Sus method accounted for the vehicle suspension in the pose estimation, so its trajectory only avoided some large undulations and was smoother than that generated by TS-NoSus.

The terrain undulations become larger in the environments shown in Figure 15b,c and there were even non-traversable areas (or called obstacles) in the latter environment. The NoTS method, which did not consider the terrain shape, calculated two nearly “straight” trajectories in these two environments. However, these trajectory were not really straight and longer than the trajectories generated by TS-NoSus and TS-Sus, because the *z*-coordinate along these “straight” trajectories changed dramatically (according to Figure 16d,g). Recall that the trajectory smoothness was computed using Equation (33). Therefore, the trajectories generated by the NoTS method were not smooth in these two environments. Besides, the TS-Sus trajectories were still shorter and smoother than the TS-NoSus trajectories in these two environments (according to Table 5). In summary, the costs of the TS-Sus trajectories were the lowest, and the runtime of TS-Sus was only a little longer than that of NoTS.

### 5.2. Real-World Experiments

**Figure 17:**
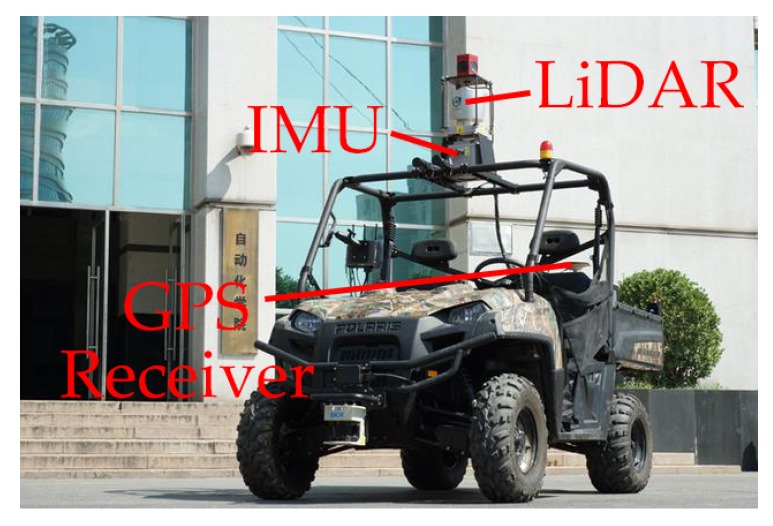
The vehicle used in experiments on rough terrain.

**Figure 18:**
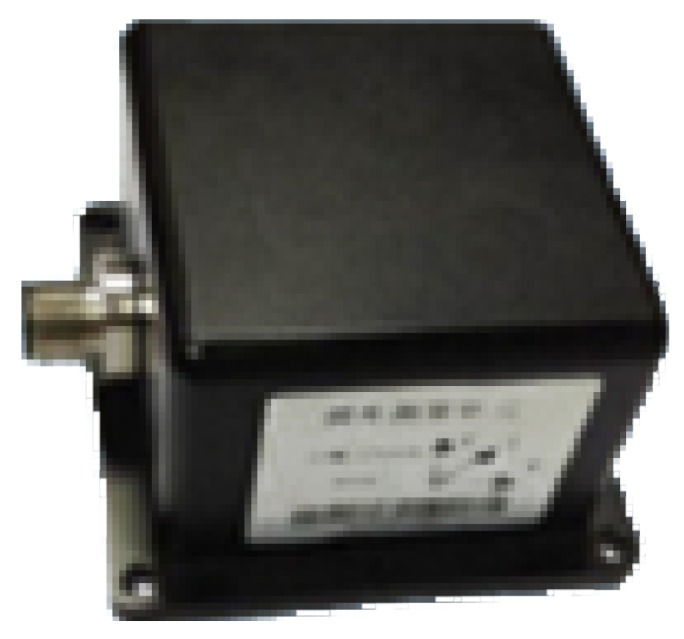
Inertial Measurement Unit (IMU).

In the real-world experiments, the point clouds of real terrain surfaces were generated by a real HDL-32E LiDAR. An all-terrain vehicle developed by Polaris (shown in Figure 17) was used to test our approaches. Besides, an inertial measurement unit (IMU) was combined with the global positioning system (GPS) to measure the roll and the pitch of the vehicle, which would be used to compute the errors of different pose estimation methods. The IMU and its specifications are shown in Figure 18 and Table 6, respectively. The GPS receiver is equipped with NT1065 “Nomada” and bladeRF as the RF front end. They can simultaneously receive various GNSS satellite signals, including GPS (L1, L2, L3, L5). The IMU is similar to an odometer, whose error increases as time goes on. This accumulated error can be decreased using GPS. The IMU can generate continuous measurements and is more robust in non-planar environments than other odometers. Therefore, the combination of the IMU and the GPS is suitable for obtaining the ground truths of the vehicle’s roll and pitch.

**Figure 19:**
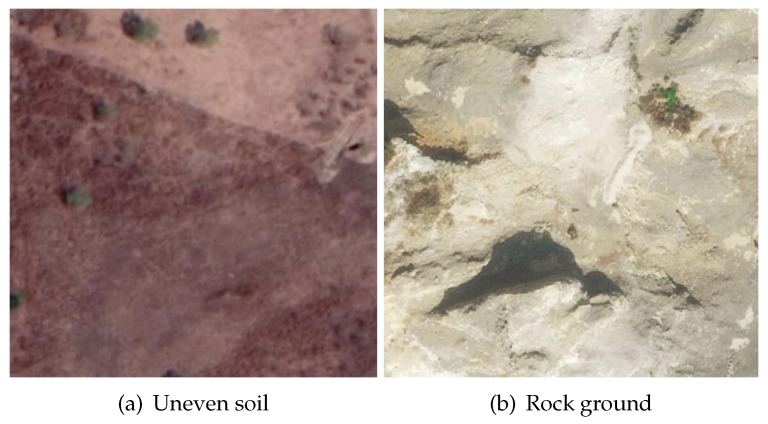
Non-planar environments in the real world.

Figure 19a,b show a piece of uneven soil and a piece of rock ground, respectively. The terrain shape of the latter is more complex than that of the former. The vehicle was driven in the above two environments, and the SE(3) pose of the vehicle was recorded at a fixed frequency. Then, different pose estimation algorithms were performed based on the planar positions and the yaws of all the recorded poses. Table 7 shows the errors of the three different pose estimation methods. Typically, the errors are larger when the vehicle runs on a more complex terrain surface. The results show that the proposed method (PC-Sus) outperformed Kin-GP-VE and DEM-Kin (by approximately 36% and 49%).

**Figure 20:**
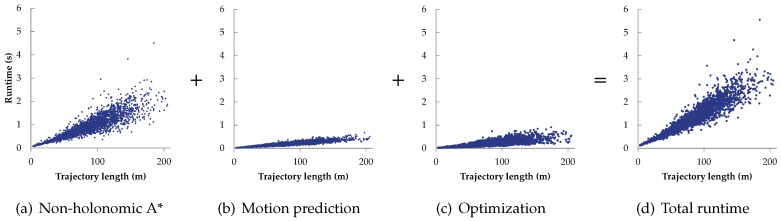
The statistic evaluation of the computational complexities.

To analyze the computational complexities of the different stages of the proposed trajectory planning approach, an experiment comprising 3000 different random planning queries (3000 pairs of start and goal poses) was conducted. For this experiment, the point clouds of the non-planar environments shown in Figure 19a,b were used. Each planning query was randomly set in one of these two environments. The results are shown in Figure 20. In all the three stages, the runtime and its variance increase approximately proportionally with the length of the planned trajectory. On average, the non-holonomic A* search is the most computationally expensive.

**Figure 21:**
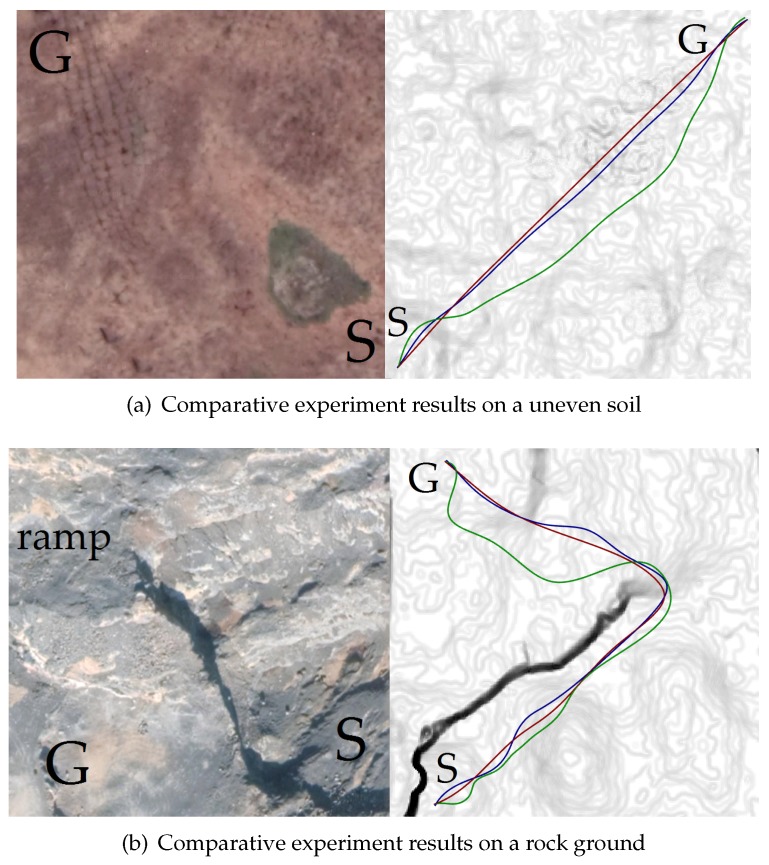
The trajectories generated by NoTS (red), TS-NoSus (green), and TS-Sus (blue).

**Figure 22:**
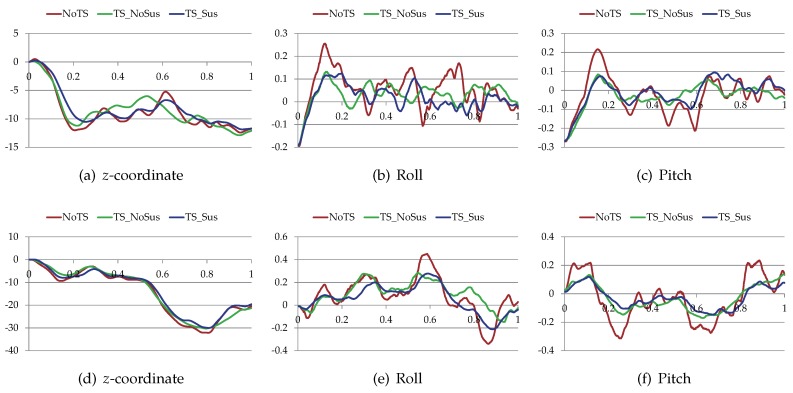
The *z*-coordinate, the roll, and the pitch of the vehicle along the paths and the trajectories. (**a**–**c**) The vehicle state along the trajectories shown in Figure 21a; (**d**–**f**) The vehicle state along the trajectories shown in Figure 21b.

Next, three different trajectory planning methods (NoTS, TS-NoSus, and the proposed TS-Sus) were compared in the real-world experiments. Figure 21a shows a piece of soil whose terrain undulations are small. In this environment, the TS-NoSus method computed a trajectory that avoided these small terrain undulations. However, the small terrain undulations often have little impact on the traversability of the vehicle that has a suspension system. Therefore, the propose TS-Sus method generated a trajectory that only avoided some large undulations. As a result, the traversability of the TS-Sus trajectory is nearly the same as that of the TS-NoSus trajectory (according to Figure 22a–c, and Table 8), but the TS-Sus trajectory is much smoother than the TS-NoSus trajectory (according to Table 8).

Figure 21b shows a piece of rock ground that has a ramp and many large terrain undulations. In this environment, the NoTS method computed a trajectory that only avoided the non-traversable areas (or called obstacles). The roll and the pitch along this trajectory were larger than those along the trajectories generated by the other two planners (according to Figure 22e,f). As a result, the traversability of this trajectory was smaller than the traversabilities of the other two trajectories (according to Table 8). In conclusion, the total costs of the trajectories generated by the proposed planner were the lowest in the comparative experiments. The runtime of our planner was a little longer than that of the NoTS planner, because our planner spent more time on processing the information of terrain shapes.

**Figure 23:**
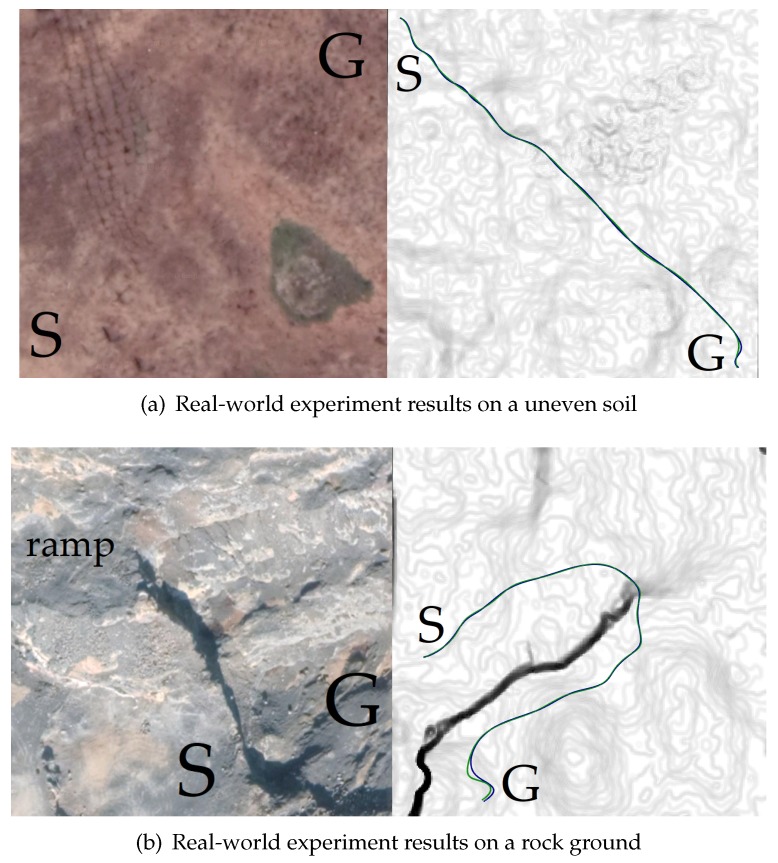
The trajectories (green) after optimization and the real trajectories (blue) of the vehicle in the real world.

At last, Figure 23 shows the trajectories (green) after optimization and the real trajectories (blue) of the vehicle in the real world. The blue trajectories were generated by making the vehicle track the green trajectories. Table 9 shows the performance comparison of these trajectories. The results show that there was little difference between the optimized trajectories (green) and the real trajectories (blue). This implies that the optimized trajectories were easy to be tracked by the vehicle on the real rough terrain. The reason is that planning based on the traversability can reduce the trajectory tracking error in a predictive way.

## 6. Conclusions

This paper presents a traversability assessment method and a trajectory planning method, which can be applied to generate a safe and efficient trajectory for a car-like vehicle running on a rough terrain surface. The proposed suspension-based traversability calculation enables planning dynamically feasible trajectories on different terrain surfaces. Also, computing the traversability on demand based on original LiDAR points helps us get rid of the explicit terrain reconstruction or discretization. Besides, the proposed traversability assessment method (or trajectory planning method) is a general approach that can be used in any other navigation system of a ground mobile robot.

The proposed method has been tested and analyzed in both the simulation and the real-world experiments. According to the experimental results, the error of our pose estimation method, which accounts for the vehicle suspension model, was smaller than the other methods that ignore the suspension model. In a non-planar environment with various terrain shapes, the proposed traversability assessment method was validated by showing a heat map colored by the traversability. Besides, the different stages of the proposed planner were analyzed in terms of the trajectory costs and the computational complexities. The results indicate that the optimization based on the suspension model could make the trajectory smoother and easier to be tracked. Finally, the proposed planner was compared with some other state-of-the-art planners in various non-planar environments. In these comparative experiments, the proposed planner showed a better generalization ability to compute trajectories with lower costs on both simple and complex terrain surfaces.

In the future, the traversability will be calculated based on not only the geometric information but also the semantic information of rough terrain surfaces. The semantic-based traversability can enable the trajectory planner to navigate a vehicle more intelligently, which needs techniques to perform the semantic segmentation of 3D point clouds from laser sensors or images from visual sensors.

## Figures and Tables

**Table 1:** The specifications of the LiDARs.

Parameter	HDL-64E S2	HDL-32E
Distance accuracy	<2 cm	<2 cm
Measurement range	50 m for pavement and 120 m for cars and foliage	70 m
Vertical field of view	$+2.0^{\circ }$ to $-24.8^{\circ }$	$+10.7^{\circ }$ to $-30.7^{\circ }$
Vertical angular resolution	$0.4^{\circ }$	$1.33^{\circ }$
Horizontal angular resolution	$0.09^{\circ }$	$0.16^{\circ }$
# Points per second	1,333,000	700,000

**Table 2:** The definitions of the constants used in the vehicle suspension models.

Notation	Definition
$m_{s}$	Mass of sprung
$m_{u_{f}}$	Mass of front unsprung
$m_{u_{b}}$	Mass of back unsprung
$I_{x}$	Roll axis moment of inertia
$I_{y}$	Pitch axis moment of inertia
$k_{t_{f}}$	Stiffness of front tire
$k_{t_{b}}$	Stiffness of back tire
$k_{f}$	Front suspension spring stiffness
$k_{b}$	Back suspension spring stiffness
$\mu_{f}$	Front suspension damping
$\mu_{b}$	Back suspension damping
$W_{f}$	Width of front sprung
$W_{b}$	Width of back sprung
$L_{f}$	Length between vehicle front axle and center of gravity of sprung
$L_{b}$	Length between vehicle back axle and center of gravity of sprung

**Table 3:** The errors of the different pose estimation methods.

Terrain Surface	Method	RMSE of Roll (Rad)	RMSE of Pitch (Rad)
Figure 11a	DEM-Kin	0.0751	0.0806
	Kin-GP-VE	0.0612	0.0654
	PC-Sus	**0.0389**	**0.0405**
Figure 11b	DEM-Kin	0.0921	0.0984
	Kin-GP-VE	0.0763	0.0817
	PC-Sus	**0.0502**	**0.0535**
Improvement over DEM-Kin		46.85%	47.69%
Improvement over Kin-GP-VE		35.33%	36.30%

**Table 4:** The performance comparison of the paths and the trajectories shown in Figure 13. Note that the real trajectories were often different between different runs, so the data of the real trajectories were averaged over 50 runs.

**Terrain Surface Shown in Figure 13a**						
**Path or Trajectory**	$d_{o}$ **(m)**	τ	$t_{f}$ **(s)**	$l_{f}$ **(m)**	**Trajectory Smoothness**	**Total Cost**
Initial path	6.09	0.9099	207.09	617.48	0.0111	1.0000
Optimized trajectory	**6.53**	**0.9281**	**200.24**	**596.23**	**1.0000**	**0.2079**
Real trajectory	6.45	0.9267	201.33	598.79	0.9822	0.2190
**Terrain Surface Shown in Figure 13b**						
**Path or Trajectory**	$d_{o}$ **(m)**	τ	$t_{f}$ **(s)**	$l_{f}$ **(m)**	**Trajectory Smoothness**	**Total Cost**
Initial path	15.44	0.9329	115.03	340.60	0.4484	1.0000
Optimized trajectory	18.08	**0.9466**	**111.71**	**330.62**	**1.0000**	**0.6461**
Real trajectory	**18.72**	0.9465	111.98	331.88	0.9937	0.6526

**Table 5:** The performance comparison of the trajectories shown in Figure 15 (averaged over 50 runs).

**Terrain Surface Shown in Figure 15a**							
**Method**	$d_{o}$ **(m)**	τ	$t_{f}$ **(s)**	$l_{f}$ **(m)**	**Trajectory Smoothness**	**Total Cost**	**Runtime (s)**
NoTS	+∞	0.9318	114.53	336.08	0.7738	0.3533	**3.220**
TS-NoSus	+∞	0.9495	121.22	344.15	0.2050	1.0000	4.861
TS-Sus	+∞	**0.9497**	**112.46**	**332.87**	**1.0000**	**0.3299**	3.338
**Terrain Surface Shown in Figure 15b**							
**Method**	$d_{o}$ **(m)**	τ	$t_{f}$ **(s)**	$l_{f}$ **(m)**	**Trajectory Smoothness**	**Total Cost**	**Runtime (s)**
NoTS	+∞	0.2765	119.81	354.92	0.2714	1.0000	**3.385**
TS-NoSus	+∞	0.9708	123.82	366.95	0.5066	0.3973	4.217
TS-Sus	+∞	**0.9795**	**117.97**	**351.42**	**1.0000**	**0.2880**	3.782
**Terrain Surface Shown in Figure 15c**							
**Method**	$d_{o}$ **(m)**	τ	$t_{f}$ **(s)**	$l_{f}$ **(m)**	**Trajectory Smoothness**	**Total Cost**	**Runtime (s)**
NoTS	6.08	0.3461	127.96	379.39	0.2966	1.0000	**3.617**
TS-NoSus	11.69	**0.9317**	152.22	452.17	0.7318	0.4407	4.418
TS-Sus	**12.82**	0.9296	**124.65**	**378.46**	**1.0000**	**0.3748**	3.730

**Table 6:** The specifications of the IMU.

Measurement Range (Roll, Pitch)	Accuracy (Roll, Pitch)	Resolution (Roll, Pitch)	Bandwidth
$-180.0^{\circ }$ to $180.0^{\circ }$	$<0.2^{\circ }$	$0.01^{\circ }$	300 Hz

**Table 7:** The errors of the different pose estimation methods.

Non-Planar Environment	Method	RMSE of Roll (Rad)	RMSE of Pitch (Rad)
Figure 19a	DEM-Kin	0.0763	0.0819
	Kin-GP-VE	0.0605	0.0640
	PC-Sus	**0.0343**	**0.0367**
Figure 19b	DEM-Kin	0.1104	0.1196
	Kin-GP-VE	0.0879	0.0963
	PC-Sus	**0.0617**	**0.0662**
Improvement over DEM-Kin		49.58%	49.92%
Improvement over Kin-GP-VE		36.56%	36.96%

**Table 8:** The performance comparison of the trajectories shown in Figure 21 (averaged over 50 runs).

**Non-Planar Environment Shown in Figure 21a**							
**Method**	$d_{o}$ **(m)**	τ	$t_{f}$ **(s)**	$l_{f}$ **(m)**	**Trajectory Smoothness**	**Total Cost**	**Runtime (s)**
NoTS	+∞	0.9046	60.47	178.57	0.9970	0.6944	**1.489**
TS-NoSus	+∞	**0.9185**	61.81	183.18	0.4490	1.0000	2.334
TS-Sus	+∞	0.9174	**60.04**	**177.68**	**1.0000**	**0.5176**	1.519
**Non-Planar Environment Shown in Figure 21b**							
**Method**	$d_{o}$ **(m)**	τ	$t_{f}$ **(s)**	$l_{f}$ **(m)**	**Trajectory Smoothness**	**Total Cost**	**Runtime (s)**
NoTS	13.45	0.5165	**69.09**	**196.83**	0.9982	1.0000	**3.103**
TS-NoSus	14.33	0.9155	78.05	222.62	0.8409	0.7358	4.840
TS-Sus	**14.51**	**0.9165**	70.54	199.08	**1.0000**	**0.6435**	3.174

**Table 9:** The performance comparison of the trajectories shown in Figure 23. Note that the real trajectories were often different between different runs, so the data of the real trajectories were averaged over 50 runs.

**Terrain Surface Shown in Figure 23a**						
**Trajectory**	$d_{o}$ **(m)**	τ	$t_{f}$ **(s)**	$l_{f}$ **(m)**	**Trajectory Smoothness**	**Total Cost**
Optimized trajectory	+∞	**0.9165**	**60.93**	**180.28**	**1.0000**	**0.9797**
Real trajectory	+∞	0.9128	61.02	180.65	0.9791	1.0000
**Terrain Surface Shown in Figure 23b**						
**Trajectory**	$d_{o}$ **(m)**	τ	$t_{f}$ **(s)**	$l_{f}$ **(m)**	**Trajectory Smoothness**	**Total Cost**
Optimized trajectory	**6.44**	**0.9143**	68.04	194.59	**1.0000**	**0.9816**
Real trajectory	6.29	0.9085	**67.92**	**194.02**	0.9804	1.0000

## Abbreviations

The following abbreviations are used in this manuscript:

UGV	Unmanned Ground Vehicle
LiDAR	Light Detection and Ranging
DEM	Digital Elevation Map
ROS	Robot Operating System
GP	Gaussian Process
VE	Vehicle Experience
RMSE	Root Mean Squared Error
TS	Terrain Shape
IMU	Inertial Measurement Unit
GPS	Global Positioning System
